# A bibliometric and text-mining analysis of lipidomics and metabolomics in human disease

**DOI:** 10.3389/fphys.2026.1727465

**Published:** 2026-05-19

**Authors:** Alejandro I. Trejo-Castro, Luis Martín Marín-Obispo, Diego Carrion-Alvarez, Shirley Mora-Godínez, Antonio Martinez-Torteya, Carmen Hernández-Brenes, Emmanuel Martinez-Ledesma

**Affiliations:** 1 School of Medicine and Health Sciences, Tecnologico de Monterrey, Monterrey, Mexico; 2 Institute for Obesity Research, Tecnologico de Monterrey, Monterrey, Mexico; 3 Internal Medicine Department, Instituto de Seguridad y Servicios Sociales de los Trabajadores del Estado (ISSSTE) Hospital Regional Monterrey, Monterrey, Mexico; 4 School of Engineering and Technology, Universidad de Monterrey, San Pedro Garza García, Mexico; 5 School of Engineering and Sciences, Tecnologico de Monterrey, Monterrey, Mexico

**Keywords:** bibliometric analysis, bioinformatics, disease diagnosis, lipidomics, metabolomics, multi-omics integration, science mapping, text mining

## Abstract

**Introduction:**

Lipidomics and metabolomics have become key approaches for understanding and diagnosing human diseases, including type 2 diabetes, Alzheimer’s disease, cancer, and kidney dysfunction. This study provides a comprehensive overview of the evolution of these disciplines through a bibliometric and text-mining analysis of scientific production from 2004 to 2024, based on data from Scopus and validated through a multi-database comparative approach.

**Methods:**

A total of 9,628 articles were harmonized and analyzed using Bibliometrix, Scimago Graphica, OpenRefine, and custom R scripts to identify the most productive journals, authors, countries, and institutions, and to map thematic structures and keyword dynamics. Beyond traditional bibliometric indicators, our integrative approach combined quantitative trends with semantic and conceptual mapping to trace the methodological and translational evolution of the field. To ensure robustness and generalizability, equivalent searches were conducted in the Web of Science Core Collection and PubMed, and cross-database validation assessed concordance in journal and country rankings, as well as temporal and thematic trends.

**Results:**

The field shows rapid expansion, with an annual growth rate of 32.6%. The United States and China lead global output, followed by major European contributors. Core topics include Alzheimer’s disease, obesity, and breast cancer, while emerging areas focus on artificial intelligence, multi-omics integration, and Mendelian randomization. Analytical methodologies such as liquid chromatography-mass spectrometry, gas chromatography-mass spectrometry, and nuclear magnetic resonance, together with metabolic diseases, remain central to the field. In contrast, niche themes such as microbiota-COVID-19 interactions and oxidative stress-cancer associations represent emerging interdisciplinary bridges.

**Discussion:**

Overall, lipidomics and metabolomics are evolving toward integrative and computational frameworks with strong diagnostic potential, underscoring the need for validated biomarkers, standardized data pipelines, and open repositories to enable clinical translation.

## Graphical Abstract

**Figure:**
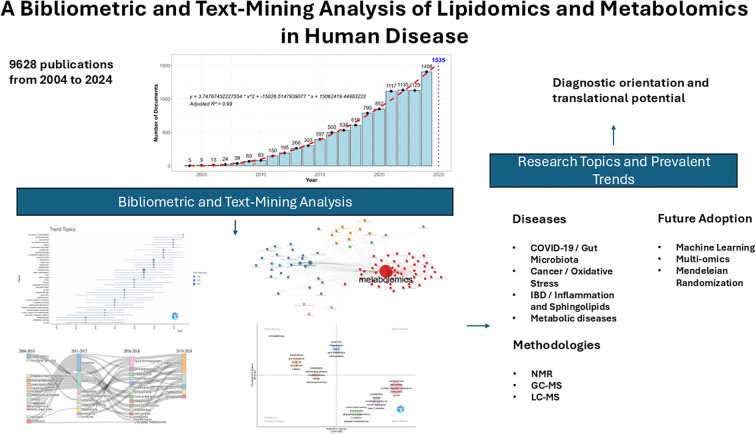

## 1 Introduction

Metabolomics systematically studies metabolites, the chemical compounds produced, used, or transformed during cellular metabolism. Metabolites, such as amino acids, fatty acids, and carbohydrates, are the downstream end products of biological processes and can function as biomarkers for clinical diagnosis (Patti et al., 2012). In addition, metabolomics can elucidate metabolite dynamics, composition, interactions, and changes in response to external interventions such as drugs, diet, or environmental conditions (Pang and Hu, 2023). These shifts can directly impact within cells, tissues and biofluids, underscoring the role of metabolomics in linking human physiology, lipid metabolism, and nutrition (Johnson et al., 2016). On the other hand, lipids are a diverse group of metabolites that serve as structural components of cell membranes, sources of energy storage, and intermediates in signaling pathways (Han and Gross, 2022).

Like genes and proteins, metabolites can also serve as signatures of biochemical activity, facilitating their correlation with disease phenotypes. Remarkably, a single mutation in a gene can affect several metabolic pathways, thereby exerting a functional influence on distinct cellular processes (Gieger et al., 2008). Nowadays, multi-omics studies scrutinize the human lipidome and/or metabolome to facilitate the development of personalized treatments, for example, by monitoring changes in metabolites and lipid levels relative to genetics or epigenetics (Astarita et al., 2023). Beyond discerning the coordination of each gene with its molecular function to promote human health, contemporary research aims to elucidate the relationship between nutrients, host metabolism, and gut microbiota, to achieve a more comprehensive understanding of biological processes and their influence on phenotypes and diseases (Orešič, 2009).

Although the terms lipidomics and metabolomics gained prominence in the early 2000s, the proposition that the quantitative analysis of metabolites and chemical constituents could be beneficial to monitor a patient’s health can be traced back several decades (Gross, 2017; Hasin et al., 2017). The advent of mass spectrometry in the early 20th century, coupled with the development of separation techniques such as gas chromatography (GC) and liquid chromatography (LC), enabled researchers such as Nobel laureate Linus Pauling and his colleague Arthur B. Robinson in 1971 to profile breath and urine vapor with the following premise: *“The thorough quantitative analysis of body fluids might permit differential diagnosis of many diseases in a more effective way than is possible at the present time”* (Pauling et al., 1971).

Lipidomics and metabolomics have become increasingly relevant for disease diagnosis. Specific metabolites, including isoleucine, leucine, valine, tyrosine, and phenylalanine, are associated with insulin resistance and β-cell function before the onset of type 2 diabetes (Wang et al., 2011). The role of lipids in disease ranges from dysregulation of the plasma lipidome in Alzheimer’s disease (Liu et al., 2021) to new research showing promising results with mass spectrometry (MS) for faster diagnosis of prostate cancer (Buszewska-Forajta et al., 2021) or improving the diagnosis and prevention of kidney function (Baek et al., 2022). Moreover, researchers have investigated lipidomic and metabolomic signatures in COVID-19 patients, showing that, despite clinical heterogeneity, patients consistently display distinct profiles when compared with healthy controls. In addition, analyses before and after tocilizumab treatment revealed a partial reversion of these alterations, suggesting that lipidomic and metabolomic profiling may help monitor therapeutic effects (Meoni et al., 2021).

Additionally, bibliometric studies are essential for monitoring the growth of research in a field. These studies enable the analysis and prediction of trends, including the estimation of the extent, timing, and participants in specific research areas (Kokol et al., 2021). These studies offer a comprehensive overview and facilitate collaboration among current researchers by highlighting analogous projects. There are two types of bibliometric analysis: performance analysis and science mapping. The former examines the contributions of authors, countries, institutions, and journals. The science mapping examines the relationships between these constituents to determine the social, intellectual, and conceptual structures of the topic of interest (Donthu et al., 2021).

The utilization of lipidomics and metabolomics in the diagnostic process has garnered significant attention, as evidenced by multiple bibliometric studies addressing specific disease contexts (see below). However, lipidomics and metabolomics encompass a broad spectrum of medical conditions, and a preliminary analysis of existing literature revealed that no study had comprehensively examined their conceptual and translational evolution across diseases.

Recently, Li et al. (2025) published a data mining-based bibliometric study that mapped the research landscape and emerging frontiers of lipidomics from 2003 to 2024, highlighting publication growth, international collaborations, and emerging themes such as ferroptosis, gut microbiota interactions, and multi-omics integration (Li et al., 2025). Their analysis, while providing a valuable goal overview of lipidomics, did not address the diagnostic dimension or the cross-omic convergence with metabolomics. In contrast, the present study jointly examines lipidomics and metabolomics to capture their conceptual convergence, research interconnections, and diagnostic translation.

The following bibliometric studies, identified through a keyword ((Metabolomics OR Lipidomics) AND Bibliometric*) search, are notable examples in this field: metabolomics in coronary artery disease (Yu et al., 2022), research on metabolic dysfunction in polycystic ovary syndrome (Xu et al., 2023), cancer metabolic reprogramming (Yang et al., 2024), the metabolomics of osteosarcoma (Tu et al., 2024), and the relationship between exercise and metabolomics (Lv et al., 2022).

The objective of this study is to explore and characterize the global scientific production in lipidomics and metabolomics applied to disease diagnosis from the early 2000s to 2024 – a period that has seen remarkable expansion in omics-based research. We seek to map the most relevant studies, diseases addressed, and analytical strategies, as well as the collaborative and thematic evolution of these fields. Through bibliometric performance and science mapping analyses, we identify key contributors, conceptual structure, and emerging research trends shaping diagnostic innovation in lipidomics and metabolomics.

- What are the current publication and collaboration patterns, including main sources, contributors, and author networks?
- How have research topics evolved over time according to publication growth, thematic evolution, and keyword dynamics?
- Which authors, institutions, and countries have been most productive or influential?
- Which diseases, analytical platforms, and concepts dominate current research?
- Which emerging themes and conceptual connections signal a transition toward clinical translation and precision medicine?

Ultimately, this study aims to provide a comprehensive overview of how lipidomics and metabolomics have evolved as diagnostic and translational sciences, highlighting disease contexts, analytical methods, and research collaborations that are driving the integration of these omics into precision medicine.

## 2 Materials and methods

### 2.1 Data source and search strategy

Scopus was used as the primary data source due to its broad coverage of high-quality scientific literature in health and biomedical sciences. A comprehensive literature search was conducted on February 8, 2025, to identify studies addressing the role of lipidomics and metabolomics in human disease-related contexts.

The search strategy was designed to retrieve original research articles focused on human biomedical studies while excluding publications based on animal or plant models and those belonging to non-biomedical disciplines. Only articles written in English and published between 2004 and 2024 were considered. As data collection occurred in early 2025, publications from later in that year may not have been fully indexed at the time of retrieval.

The search yielded a total of 9,728 records, which constituted the primary dataset for subsequent bibliometric analyses. A detailed description of the search query, inclusion and exclusion criteria, data handling procedures, and data analysis considerations are provided in the **Supplementary File 1**.

**Figure 1:**
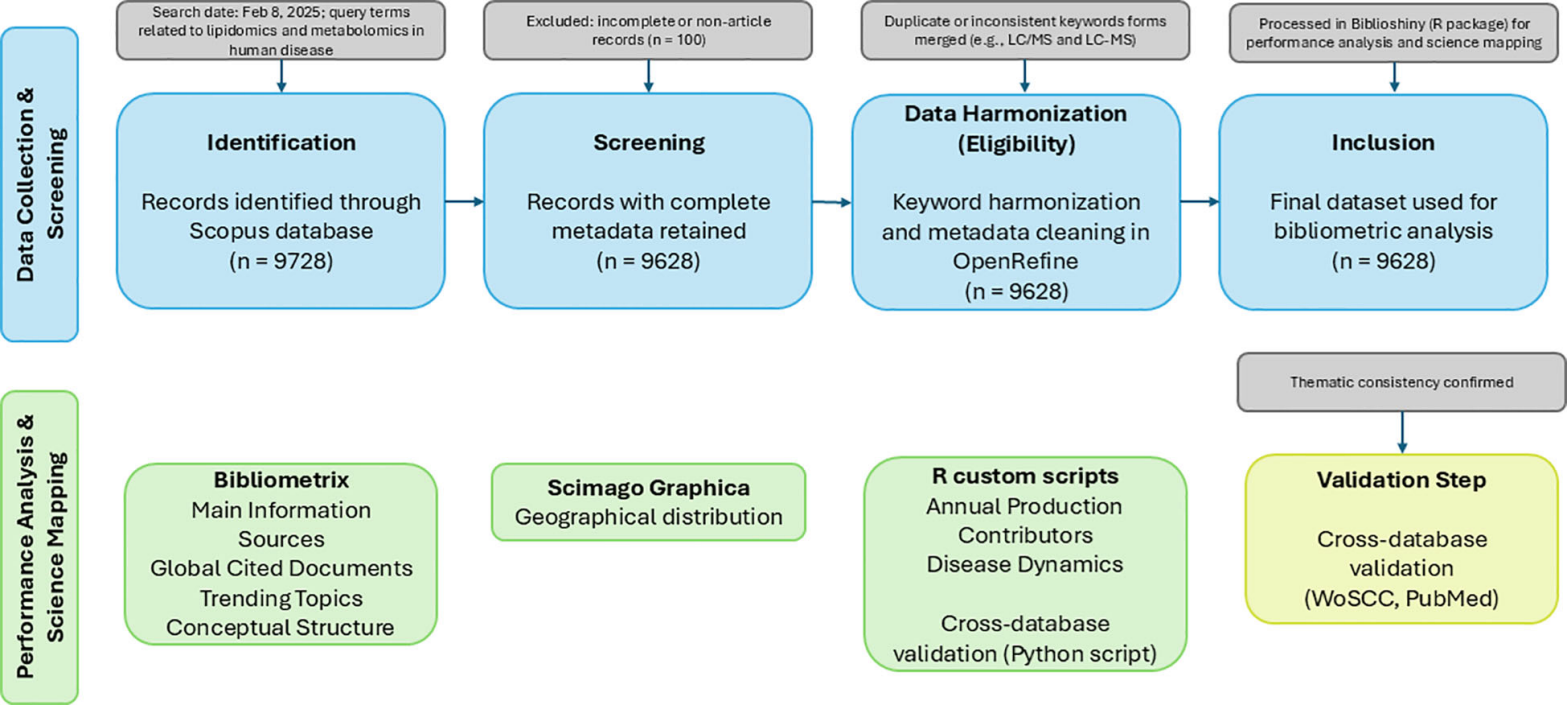
PRISMA and bibliometric analysis workflow steps.

**Figure 1** illustrates the systematic identification, screening, and inclusion of records retrieved from Scopus, followed by data cleaning, harmonization, and visualization workflows, following PRISMA principles adapted from bibliometric studies (Ahmed et al., 2023; Murthy et al., 2025). The raw and processed datasets derived from Scopus are provided in **Supplementary Files 2**, **3**.

### 2.2 Data filtering

Data filtering was performed using the Biblioshiny interface of the Bibliometrix package to retain only records with complete bibliographic metadata (Aria and Cuccurullo, 2017; Derviş, 2020). From the initial set of 9,728 retrieved articles, a total of 9,628 records met this criterion and were retained for subsequent analyses.

The final curated dataset used in the analyses is provided in **Supplementary File 4**, together with a metadata completeness report generated by Biblioshiny (**Supplementary File 5**).

### 2.3 Data harmonization

Bibliometric analyses require a preliminary data harmonization step to address inconsistencies and variations in bibliographic metadata, which can affect the reliability of thematic analyses. In this study, harmonization focused on author’s keywords, as these terms best capture the conceptual content of each publication.

To unify semantically equivalent terms expressed in different lexical forms, we applied a combined automated and manual harmonization strategy using the open-source tool OpenRefine (Ham, 2013; CS&S, 2024). Automated clustering methods were first used to group closely related keyword variants, followed by manual inspection to prevent overgeneralization and to ensure conceptual accuracy. It is noteworthy to mention that manual inspection was limited to validating automated clustering outcomes and correcting a small number of clearly overgeneralized cases, accounting for less than 1% of the total keyword terms.

This process allowed for the consolidation of synonymous disease names, analytical platforms, and methodological terms into standardized representations, resulting in a harmonized keyword dataset suitable for robust downstream analyses. Detailed descriptions of the harmonization procedures and outputs are provided in **Supplementary Files 6**-**8**.

### 2.4 Data analysis

Data analysis was conducted using a combination of Bibliometrix/Biblioshiny, OpenRefine, and custom R scripts to perform performance analysis and scientific mapping. Custom scripts were developed to address known limitations of automated bibliometric tools, particularly for author disambiguation and country level productivity metrics.

To avoid inflation of national research output caused by multi-author publications, scientific production was quantified at the document level rather than by author counts. Scimago Graphica was used for graphical representation of selected indicators (Hassan-Montero et al., 2022). Document-level analyses were performed to address research questions related to disease representation and thematic focus. Author keywords were analyzed to identify the most frequently studied diseases, which were grouped into broader conceptual categories using a curated disease dictionary. Temporal analyses were conducted to examine shifts in research emphasis over time, including the emergence of pandemic-related research after 2020.

Journal performance was assessed using Scopus-derived indicators, including CiteScore (CS) (Teixeira da Silva and Memon, 2017), SCImago Journal Rank (SJR) (Guz and Rushchitsky, 2009), and Source Normalized Impact per Paper (SNIP) (Moed, 2010), to provide a multidimensional view of source influence across study areas.

The PICOS framework was not applied, as this study focuses on bibliometric and text-mining analyses rather than on structured clinical outcomes typical of systematic reviews.

### 2.5 Multi-database validation

To assess the robustness and comprehensiveness of the results obtained from the Scopus dataset, additional searches were conducted in the Web of Science Core Collection (WoSCC) and PubMed. Equivalent search strategies, based on the same keywords, Boolean operators, and a time frame restricted to 2004-2024, were adapted to the syntax of each database. Inclusion and exclusion criteria were applied consistently across all sources to ensure comparability.

For each supplementary database, the same general analytical framework was applied, including verification of metadata completeness. Keyword harmonization was not performed, as differences in indexing terminology across databases were expected. Analyses focused on total record counts, annual publication trends, and country-level productivity based on publication and corresponding author information.

Cross-database comparisons emphasized conceptual consistency rather than exact numerical agreement. Concordance in temporal trends was evaluated using linear regression analyses of annual publication counts. This approach allowed us to determine whether thematic focus, geographical distribution, and temporal patterns identified in the Scopus-based analysis were reproducible across other major bibliographic sources.

This validation framework follows recent bibliometric studies (Bai et al., 2025) and aligns with methodological recommendations for cross-database comparison and data cleaning (Lim et al., 2024).

## 3 Results

### 3.1 Overview

#### 3.1.1 Main information

Our preliminary analysis of the 9,628 documents reveals a wealth of insightful information regarding the evolution of lipidomics and metabolomics in disease diagnosis. **Table 1** summarizes key descriptive statistics of the dataset and highlights fundamental trends in the literature. We observed a notable annual growth rate of 32.58%, which aligns with the accelerated expansion of research in lipidomics and metabolomics for disease diagnosis and personalized healthcare. This metric demonstrates the growing recognition of the clinical and research relevance of lipidomics and metabolomics.

**Table 1:** Summary of descriptive information on the dataset collection found from 2004 to 2024.

Feature	Explanation	Count
Main information about data		
Documents	Total number of scientific publications	9628
Sources	The frequency distribution of sources such as journals and books	1891
Annual growth rate %	The average increase in the number of documents over a year	32.58
Document average age	Average age of a document given in years	5.47
Average citations per doc	The average number of quotes in each article	29.55
Document contents		
Keywords plus (ID)	Total number of words or phrases that frequently appear in the title of an article’s references	37177
Author’s keywords (DE)	Total number of keywords	14302
Authors		
Authors	Total number of authors	65979
Authors of single-authored docs	The number of single authors per article	83
Authors collaboration		
Single-authored docs	Total number of single-authored documents	85
Co-authors per doc	The average number of co-authors in each document	9.91
International Co-authorships %	The average number of articles with international collaboration	31.38

We also observed that the documents have an average age of 5.47 years over the period (2004-2024), indicating a recent surge in publications. This trend underscores sustained research activity and highlights the increasing momentum of the field. The identification of 14,302 author keywords underlines the comprehensive thematic scope, encompassing the application of lipidomics and metabolomics in diagnostics. These keywords provide the foundation for further analysis and encapsulate the core concepts and thematic directions emphasized in current research.

Our analysis of collaboration trends offers valuable insights. The data show that three out of every ten articles (31.38%) resulted from international collaborations with an average of 9.91 co-authors per publication. This high degree of co-authorship reflects the multidisciplinary nature of lipidomics and metabolomics. These fields draw on expertise from medical and biological sciences, technological developments (e.g., LC and GC -MS), systems biology, and bioinformatics. By integrating these disciplines, researchers have fostered global cooperation and enabled significant advancements in high-throughput analytical techniques and computational modeling, both of which are essential for deciphering complex biological systems.

#### 3.1.2 Annual scientific production

**Figure 2:**
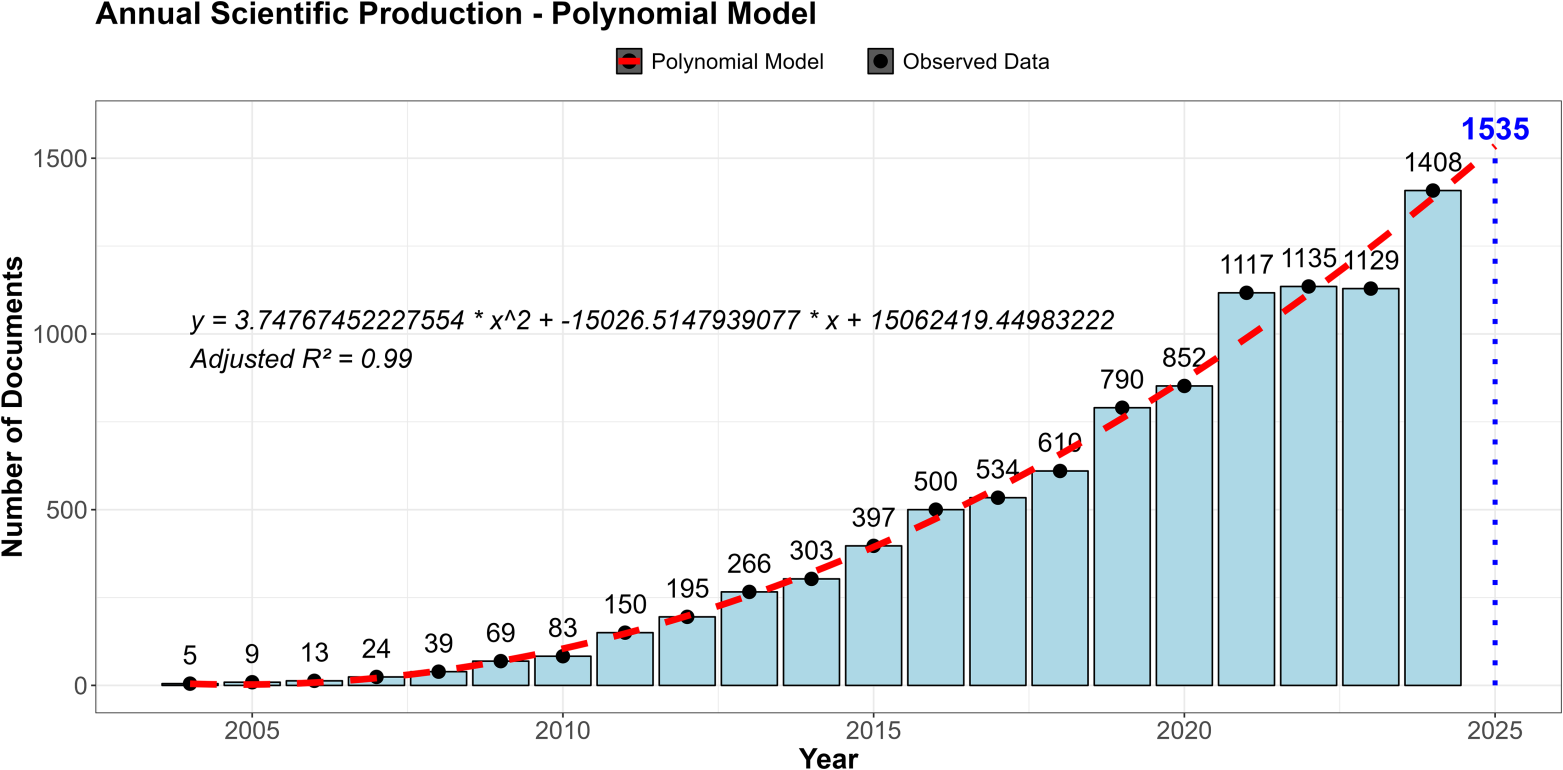
Annual distribution of scientific publications and polynomial forecast for 2025 in lipidomics and metabolomics for disease diagnosis.

**Figure 2** illustrates the progression of annual scientific output in the domains of lipidomics, and metabolomics as applied to disease diagnosis. The data reveals a continuous growth since 2004, with a more pronounced upward trajectory commencing in 2010 that signaled the consolidation of this field of study. A notable milestone was attained in 2011 when the number of publications surpassed 100 for the first time. A decade later, in 2021, publications exceeded 1,000, further solidifying the field’s expanding relevance. In 2024, output reached its highest level to date, indicating sustained interest within the scientific community.

To analyze the evolution of scientific production and project future trends, we fitted a second-degree polynomial regression model (**Figure 2**). This model captures the variability in acceleration and deceleration of growth over time, providing a more accurate representation of the annual growth rate compared to an exponential model. Our fitted model achieved an adjusted R^2^ of 0.99, indicating excellent agreement with the observed data. Because this feature measures the relative predictive power of the polynomial regression model, a value close to 1 indicates an excellent fit to the observed data. The polynomial model also indicated a growth trend but shows a slowdown in recent years (2021-2023).

Projections based on the model estimated that researchers working on lipidomics and metabolomics would publish approximately 1,535 scientific papers in 2025. However, updated Scopus data reviewed up to mid-April 2026 identified 1,788 publications for the year 2025. This discrepancy suggests that the recent growth of the field may be accelerating beyond the trend captured by the model, reinforcing the overall conclusion of sustained and expanding research activity in lipidomics and metabolomics for diagnosis.

We provide the annual production data in **Supplementary File 9**.

### 3.2 Sources

The document’s source refers to the title of a journal, book, or conference proceeding. In this bibliometric analysis, we evaluated the impact and productivity of sources in lipidomics, and metabolomics applied to disease diagnosis following Bradford’s Law (Brookes, 1969). This identifies the most relevant sources within a research domain. We found that the core zone of the most productive and influential periodicals includes 28 sources (1.48%), which collectively published 3,205 documents (33.29%). **Table 2** presents 20 of these 28 core sources, ranked by the number of publications (TP), along with their h-index (h), g-index (g), and m-index (m). These metrics provide a multi-dimensional view of each journal’s influence:

**Table 2:** List of journals with the highest number of publications on the subject.

Source	TP	TC	h	g	m	YFP	CS	SJR	SNIP	HP	Q
Scientific Reports	441	11795	54	80	4.154	2013	7.5	0.900	1.182	92^nd^	Q1
Metabolites	329	4162	31	45	2.385	2013	5.7	0.903	0.843	60^th^	Q2
Metabolomics	283	8293	45	78	2.143	2005	6.6	0.740	0.763	70^th^	Q2
Journal of Proteome Research	266	13197	64	103	3.200	2006	9.0	1.299	0.896	84^th^	Q1
PLoS ONE	253	13132	64	106	3.368	2007	6.2	0.839	1.084	89^th^	Q1
International Journal of Molecular Sciences	209	2145	25	34	1.923	2013	8.1	1.179	1.120	90^th^	Q1
Analytical Chemistry	150	9339	53	94	2.524	2005	12.1	1.621	1.274	91^st^	Q1
Frontiers in Immunology	98	1296	19	31	2.375	2018	9.8	1.868	1.193	77^th^	Q1
Journal of Pharmaceutical and Biomedical Analysis	88	1910	25	39	1.471	2009	6.7	0.584	0.927	76^th^	Q1
Clinica Chimica Acta	86	1837	26	40	1.529	2009	10.1	1.016	1.086	86^th^	Q1
Journal of Chromatography B	83	2950	29	52	1.526	2007	5.6	0.539	0.835	65^th^	Q2
Analytica Chimica Acta	83	2359	28	46	1.556	2008	10.4	0.998	1.067	91^st^	Q1
Analytical and Bioanalytical Chemistry	81	2677	32	49	1.684	2007	8.0	0.686	0.859	83^rd^	Q1
Journal of Lipid Research	59	3272	29	57	1.813	2010	11.1	2.090	1.394	89^th^	Q1
Frontiers in Endocrinology	58	495	12	19	1.333	2017	5.7	1.240	1.122	60^th^	Q2
eBiomedicine	54	1559	24	38	2.400	2016	17.7	3.193	1.728	97^th^	Q1
Journal of Clinical Endocrinology and Metabolism	53	1725	23	40	1.533	2011	11.4	1.899	1.574	90^th^	Q1
Journal of Translational Medicine	53	1006	20	30	1.667	2014	10.0	1.611	1.234	94^th^	Q1
Cancers	51	765	16	25	2.286	2019	8.0	1.391	1.030	79^th^	Q1
Journal of Chromatography A	50	1824	25	42	1.471	2009	7.9	0.717	0.923	85^th^	Q1

TP, Total Publications; TC, Total Citations; h, h-index; g, g-index; m, m-index; YFP, Year of First Publication; CS, CiteScore 2023; SJR, SJR 2023; SNIP, SNIP 2023; HP, Highest Percentile; Q, Quartile.

- H-index (h): counts the number of papers (h) that have received at least h citations, capturing both productivity and impact (Hirsch, 2005).
- G-index (g): refines the h-index by requiring that the top g articles receive at least g^2^ citations, which always makes g greater than or equal to h (Egghe, 2006).
- M-index (m): divides h by the number of years since the first publication, helping evaluate emerging researchers or sources (von Bohlen und Halbach, 2011).

Our analysis highlights that the most productive journals in this area include Scientific Reports (441 documents), Metabolites (329 documents), and Metabolomics (283 documents). Regarding citation impact, the leading sources are Journal of Proteome Research (Total Citations = 13,197, h = 64, g = 103), PLoS ONE (TC = 13,132, h = 64, g = 106), and Scientific Reports (TC = 11,795, h = 54, g = 80). In terms of prestige, based on CS, SJR, and SNIP, the top journals are eBiomedicine (CS = 17.7, SJR = 3.193, SNIP = 1.728), Journal of Lipid Research (CS = 11.1, SJR = 2.090, SNIP = 1.394), and Journal of Clinical Endocrinology and Metabolism (CS = 11.4, SJR = 1.899, SNIP = 1.574).

The diversity of journals and sources underscores the highly interdisciplinary nature of lipidomics and metabolomics, putting together fields such as analytical chemistry, molecular biology, computational sciences, medicine, and clinical research. The presence of high-impact analytical journals, including Analytical Chemistry, Analytica Chimica Acta, and Journal of Chromatography B: Analytical Technologies in the Biomedical and Life Sciences, highlights the crucial role of advanced analytical techniques in metabolomics.

Journal of Lipid Research, Journal of Clinical Endocrinology and Metabolism, and Clinica Chimica Acta are biomedical and clinical journals that reflect the growing clinical relevance of lipid and metabolomic biomarkers in disease diagnosis. Similarly, Metabolites and Journal of Translational Medicine frequently publish bioinformatics and data-driven studies, while Frontiers in Endocrinology has increasingly incorporated multi-omics analyses within endocrine research.

This broad spectrum of publication venues signals the field’s shift toward interdisciplinary collaboration, leveraging cutting-edge analytical tools and computational techniques to advance biomarker discovery, personalized medicine, and disease classification in lipidomics and metabolomics.

### 3.3 Contributors

#### 3.3.1 Authors

**Table 3** presents the top 10 authors ranked by productivity, complemented with the TC, h, g, and m metrics previously applied in the source analysis. We retrieved each author’s affiliation from Scopus, using their most recent publication as a reference. Their institutions are in the United States (4), Europe (3), Australia (2), and China (1).

**Table 3:** List of authors with the highest number of publications on the subject.

Author	Affiliation	TP	TC	h	g	m	YFP
Guowang Xu	Dalian Institute of Chemical Physics Chinese Academy of Sciences, Dalian, China	74	4310	36	65	1.714	2005
Coral Barbas	Universidad CEU San Pablo, Madrid, Spain	60	1788	23	41	1.353	2009
Dean P. Jones	Emory University School of Medicine, Atlanta, United States	54	1429	22	36	1.222	2008
Oliver E. Fiehn	University of California, Davis, Davis, United States	51	3610	28	51	1.400	2006
Elaine C. Holmes	Murdoch University, Perth, Australia	49	3797	25	49	1.250	2006
Jerzy Adamski	Univerza v Ljubljani Medicinska Fakulteta, Ljubljana, Slovenia	48	4162	27	48	1.688	2010
Peter J. Meikle	Baker Heart and Diabetes Institute, Melbourne, Australia	46	2690	25	46	1.667	2011
Daniel Raftery	University of Washington, Seattle, United States	45	1735	24	41	1.263	2007
Clary B. Clish	Broad Institute, Cambridge, United States	43	5695	21	43	0.955	2004
Gabi Kastenmüller	Helmholtz Center Munich German Research Center for Environmental Health, Oberschleissheim, Germany	42	2792	22	42	1.467	2011

TP, Total Publications; TC, Total Citations; h, h-index; g, g-index; m, m-index; YFP, Year of First Publication.

Our analysis highlights Guowang Xu, from the Dalian Institute of Chemical Physics, Chinese Academy of Sciences, as the most prolific author. Xu leads not only in the number of publications but also in the h, g, and m indices, ranking second only in total citations. In contrast, Clary B. Clish, although ranked ninth by publication count, holds the highest position in total citations, underscoring the impact of his contributions.

**Figure 3:**
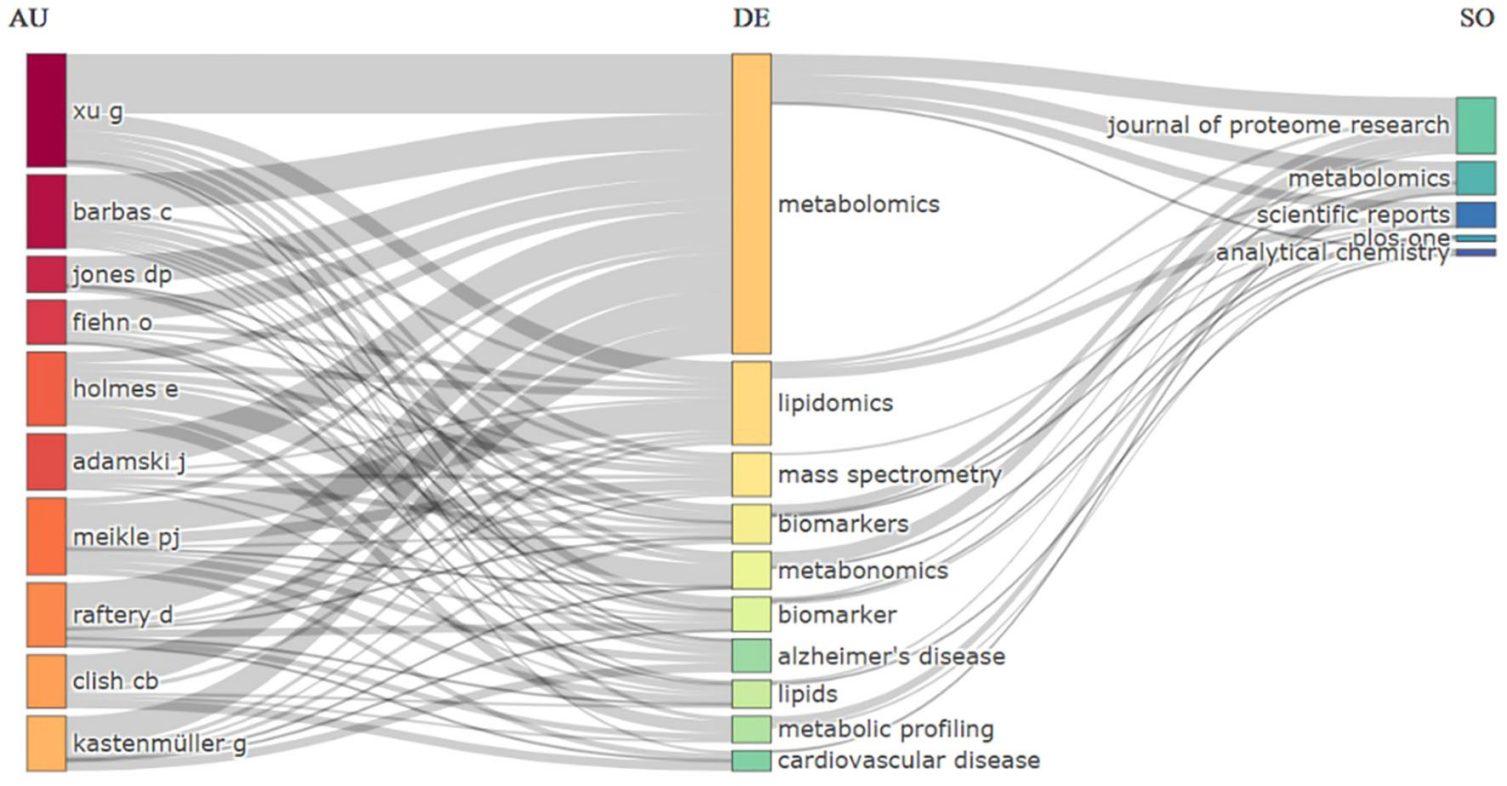
Three fields plot analysis (AU, top 10 authors; DE, author keywords; SO, source).

After evaluating the author’s performance, we extended the study with a science mapping exercise to examine the relationships between these authors, their research areas, and the journals where they published. **Figure 3** presents a three-field plot illustrating these connections. Consistent with our source analysis, these authors concentrate their work in the most productive journals. Moreover, their research keywords frequently reference Alzheimer’s disease and cardiovascular disease, highlighting the clinical relevance and thematic focus of their contribution.

To provide more depth to these metrics, we conducted a detailed analysis of the articles authored by this group of researchers. For example, Xu’s most cited article described an analysis of the follow-up of 25 patients over 12 years after recovery from SARS infection, reporting that serum metabolomic profiles showed significant differences compared with healthy controls (Wu et al., 2017). Beyond this study, Xu has also contributed to highly cited research on prostate cancer (Ren et al., 2016), hepatocellular carcinoma (Luo et al., 2018), and Parkinson’s disease (Shao et al., 2021), demonstrating the breadth of his scientific impact.

Coral Barbas authored a widely cited review that describes available validation strategies in untargeted metabolomics and recommends practical steps to develop any analytical method (Naz et al., 2014). In parallel, highly cited disease-focused studies addressed metabolic profiling in Alzheimer’s disease (González‐Domínguez et al., 2014) and acute coronary syndrome (Vallejo et al., 2009).

Other leading researchers also stand out for their contributions to both methodological advances and disease understanding. Daniel P. Jones, for example, published two influential studies on adolescents and young adults. One of these papers reported that increased lipolysis and fatty acid oxidation may aid in linking exposure to per- and polyfluoroalkyl substances (PFAS) with impaired glucose metabolism in young adults (Chen et al., 2020). The other paper showed that amino acid metabolic pathways are imbalanced in adolescents with elevated hepatic steatosis (Jin et al., 2016). Oliver Fiehn has likewise produced impactful work, including a urinary metabolomics approach for identifying kidney cancer (Kind et al., 2007), plasma metabolic profiles reflecting a glucose homeostasis in non-diabetic and obese African American women with type 2 diabetes (Fiehn et al., 2010), and a technical benchmark study validating quantitative untargeted lipidomics across nine LC-HRMS platforms (Cajka et al., 2017).

Together, these contributions illustrate the dual nature of the field, where methodological innovations in validation and quantification complement high-impact disease-focused studies. This synergy reinforces the relevance of metabolomics across both technical and clinical domains, underscoring its growing maturity as a discipline.

#### 3.3.2 Affiliations

As in the author analysis, we also report the main affiliations provided by Scopus. The top 10 institutions contributing to research in lipidomics and metabolomics for disease are: Harvard Medical School (276), Chinese Academy of Sciences (267), Ministry of Education of the People’s Republic of China (247), Imperial College London (220), Inserm-Institut national de la santé et de la recherche médicale (184), Brigham and Women’s Hospital (159), Peking Union Medical College (158), Helmholtz Center Munich German Research Center for Environmental Health (149), Instituto de Salud Carlos III (138), and Karolinska Institutet (136).

#### 3.3.3 Countries

**Table 4** shows the countries that produce the most documents on this topic. Two countries have the highest number of publications using lipidomics and metabolomics for human disease diagnosis: the United States and China. These countries also stand out by having the most articles as corresponding author (CA), single-country publications (SCP), multi-country publications (MCP), and total citations (TC). It is worth noting that from this list of 20 countries, the most collaborative countries are Finland (MCP/CA ratio = 0.702), Sweden (0.667), and Denmark (0.627). Every continent except Africa is represented, with the United States and Canada in North America, Brazil in South America, the United Kingdom, Germany, Italy, and Spain in Europe, China and Japan in Asia, and Australia in Oceania.

**Table 4:** List of countries with the highest number of publications on the subject in our collection.

Country	TP	CA	SCP	MCP	MCP/CA ratio	TC	TCCA
United States	2664	1797	1105	692	0.385	112570	75832
China	2663	2391	2018	373	0.156	53932	44010
United Kingdom	967	433	185	248	0.573	41761	18049
Germany	777	379	178	201	0.530	28051	13563
Italy	674	428	274	154	0.360	21236	11109
Spain	561	382	253	129	0.338	17629	10711
Netherlands	494	258	110	148	0.574	19388	6554
Canada	449	243	141	102	0.420	22982	15303
France	403	229	129	100	0.437	17737	6121
Japan	375	283	221	62	0.219	12132	9474
Sweden	368	174	58	116	0.667	16247	5244
Australia	345	160	74	86	0.537	12179	4618
India	302	188	131	57	0.303	8831	3150
Finland	242	121	36	85	0.702	12274	5970
Denmark	229	83	31	52	0.627	7017	1551
South Korea	229	180	147	33	0.183	5821	4203
Switzerland	221	101	43	58	0.574	10609	3948
Poland	214	136	97	39	0.287	6291	2562
Brazil	211	151	106	45	0.298	3399	2203
Austria	157	60	22	38	0.633	6323	1744

TP, Total Publications; CA, Corresponding Author; SCP, Single Country Publication; MCP, Multiple Country Publication; TC, Total Citations; TCCA, Total Citations as Corresponding Author.

**Figure 4:**
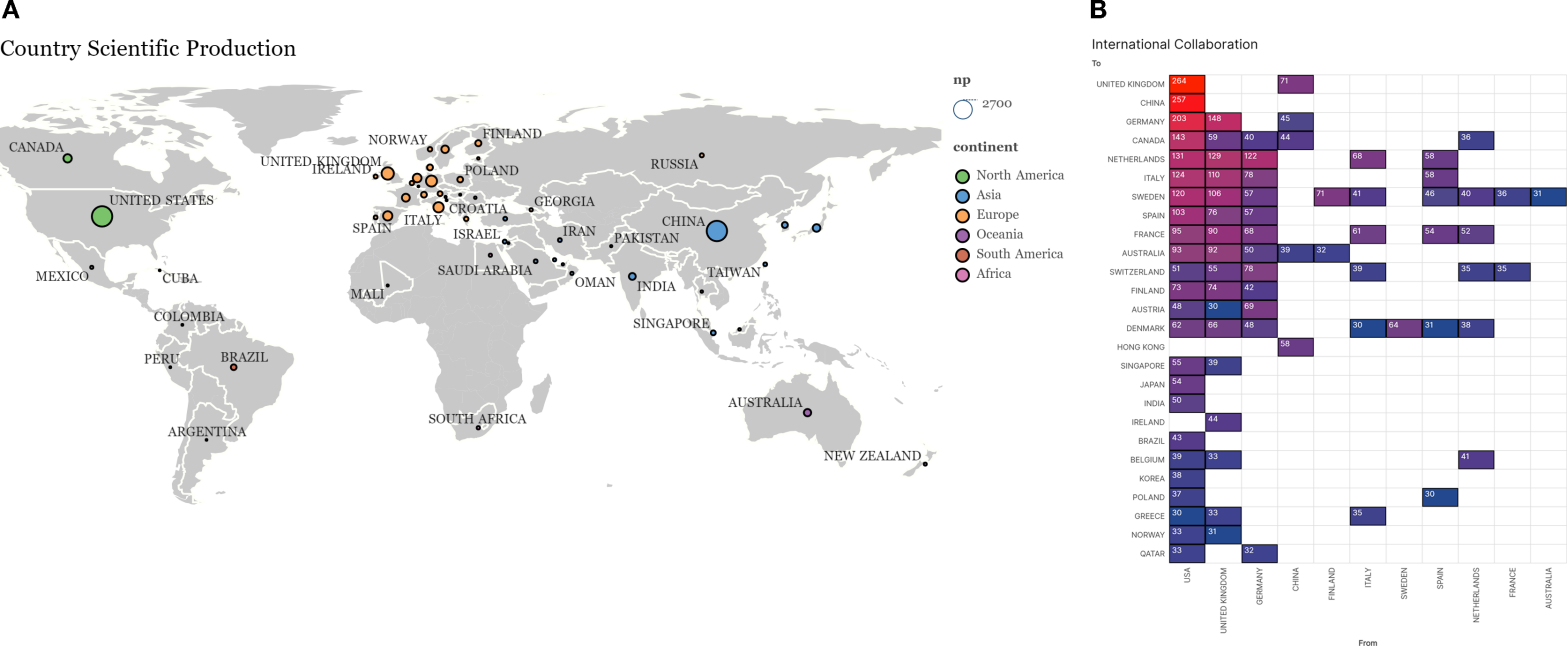
Country scientific production. **(A)** Distribution of the most productive countries on the world map with at least 20 documents. The color represents the continent, while the size of the circle is proportional to the number of publications. This image was created using Scimago Graphica. **(B)** International collaborations between countries. The number represents the total number of articles co-authored by countries. The red color represents the highest collaboration, and the blue color represents the lowest. The lowest collaboration is defined as 30.

**Figure 4A** shows the distribution of the most productive countries on the world map with at least 20 documents. With this information, it is possible to form regional research groups with international governmental organizations and the private sector to mobilize the world towards the use of lipidomics and metabolomics for human disease diagnostics. In addition to this objective, **Figure 4B** shows a matrix of those collaborations with a minimum of 30 documents between countries. The number corresponds to the total number of articles they share based on the information calculated in Biblioshiny. The country-specific results and world collaboration data are available in **Supplementary Files 10**, **11**.

### 3.4 Document analysis

#### 3.4.1 Most global cited documents

**Table 5** shows the 20 most cited articles worldwide in Scopus that are part of the collection. This information also displays the reference with the total number of citations (TC), total citations per year (TCY), and the normalized total citations (NTC). Additionally, we classified the article type into one of the following categories: method (methodology, protocols, databases, or algorithms), reviews (systematic reviews or literature reviews), and original research, which involves the application of methods in metabolomics/lipidomics studies for a specific indication or disease.

**Table 5:** Publications with the highest number of citations.

Reference	Title	Source	Article type	TC	TCY	NTC
(Wishart et al., 2018)	HMDB 4.0: the human metabolome database for 2018	Nucleic Acids Research	Method	2677	334.63	64.78
(Wishart et al., 2007)	HMDB: the Human Metabolome Database	Nucleic Acids Research	Method	2549	134.16	10.38
(Wang et al., 2011)	Metabolite profiles and the risk of developing diabetes	Nature Medicine	Original Research	2501	166.73	28.32
(Sreekumar et al., 2009)	Metabolomic profiles delineate potential role for sarcosine in prostate cancer progression	Nature	Original Research	1930	113.53	13.21
(Witwer et al., 2013)	Standardization of sample collection, isolation and analysis methods in extracellular vesicle research	Journal of Extracellular Vesicles	Method	1910	146.92	25.53
(Ajikumar et al., 2010)	Isoprenoid Pathway Optimization for Taxol Precursor Overproduction in *Escherichia coli*	Science	Original Research	1433	89.56	11.63
(Franzosa et al., 2018)	Gut microbiome structure and metabolic activity in inflammatory bowel disease	Nature Microbiology	Original Research	1213	173.29	34.59
(Gajer et al., 2012)	Temporal Dynamics of the Human Vaginal Microbiota	Science Translational Medicine	Original Research	1122	80.14	14.58
(Shen et al., 2020)	Proteomic and Metabolomic Characterization of COVID-19 Patient Sera	Cell	Original Research	1103	183.83	42.08
(Evans et al., 2009)	Integrated, nontargeted ultrahigh performance liquid chromatography/electrospray ionization tandem mass spectrometry platform for the identification and relative quantification of the small-molecule complement of biological systems	Analytical Chemistry	Method	1099	64.65	7.52
(Thévenot et al., 2015)	Analysis of the Human Adult Urinary Metabolome Variations with Age, Body Mass Index, and Gender by Implementing a Comprehensive Workflow for Univariate and OPLS Statistical Analyses	Journal of Proteome Research	Original Research	1012	92.00	18.99
(Chen et al., 2012)	Personal omics profiling reveals dynamic molecular and medical phenotypes	Cell	Method	1012	72.29	13.15
(Sakaue et al., 2021)	A cross-population atlas of genetic associations for 220 human phenotypes	Nature Genetics	Original Research	917	183.40	48.42
(Want et al., 2010)	Global metabolic profiling procedures for urine using UPLC-MS	Nature Protocols	Method	911	56.94	7.39
(Yachida et al., 2019)	Metagenomic and metabolomic analyses reveal distinct stage-specific phenotypes of the gut microbiota in colorectal cancer	Nature Medicine	Original Research	878	125.43	25.04
(Mapstone et al., 2014)	Plasma phospholipids identify antecedent memory impairment in older adults	Nature Medicine	Original Research	853	71.08	14.88
(Suhre et al., 2011)	Human metabolic individuality in biomedical and pharmaceutical research	Nature	Original Research	848	56.53	9.60
(Sugimoto et al., 2010)	Capillary electrophoresis mass spectrometry-based saliva metabolomics identified oral, breast and pancreatic cancer-specific profiles	Metabolomics	Original Research	813	50.81	6.60
(Floegel et al., 2013)	Identification of serum metabolites associated with risk of type 2 diabetes using a targeted metabolomic approach	Diabetes	Original Research	811	62.38	10.84
(Xia et al., 2013)	Translational biomarker discovery in clinical metabolomics: An introductory tutorial	Metabolomics	Method	769	59.15	10.28

TC, Total Citations; TCY, Total Citations per Year; NTC, Normalized Total Citations.

The most cited studies, *“HMDB 4.0: the human metabolome database for 2018”* (Wishart et al., 2018) and *“HMDB: the Human Metabolome Database”* (Wishart et al., 2007), describe the Human Metabolome Database (HMDB) as the standard reference resource for human metabolomic studies. The HMDB functions as a free electronic database that curates a comprehensive collection of human metabolites, including peptides, lipids, amino acids, nucleic acids, carbohydrates, organic acids, biogenic amines, vitamins, minerals, food additives, drugs, cosmetics, contaminants, and pollutants.

Each entry in the HMDB contains up to 130 data fields that integrate several types of information. These include chemical data, such as compound descriptions, synonyms, structural and physicochemical properties, reference NMR and MS spectra, and biofluid concentrations; clinical data, covering biological locations and disease associations; and molecular biology and biochemistry data, encompassing pathways, enzymes, gene sequences, SNPs, and mutation information.

In addition, HMDB links each entry to external resources, for example, KEGG, PubChem, MetaCyc, ChEBI, PDB, UniProt, and GenBank, as well as images and references. The HMDB is freely accessible at https://www.hmdb.ca/.

HDMB has expanded dramatically since its first release in 2007, which included only 2,180 molecules. The fifth version, launched in 2022, reported 217,920 annotated metabolites and introduced new tools for visualizing structures and pathways. This continuous growth positions the HMDB as a cornerstone not only for human metabolomics but also for related fields such as exposomics, nutritional sciences, biochemistry, and clinical chemistry (Wishart et al., 2022).

The third study, “Metabolite profiles and the risk of developing diabetes”, followed 2,422 normoglycemic individuals for 12 years. At baseline, the researchers measured circulating metabolites, finding that five branched-chain and aromatic amino acids showed a highly significant association with the future development of diabetes among the 201 individuals who progressed to the disease (Wang et al., 2011).

The list also includes additional studies related to other diseases. For example, researchers identified sarcosine as markedly elevated during prostate cancer progression to metastasis, and they demonstrated that it can be detected non-invasively in urine. Experimental results further showed that inhibiting the enzyme responsible for generating sarcosine from glycine attenuated prostate cancer invasion (Sreekumar et al., 2009).

Another study explored the interplay between microbiota and metabolic activity in inflammatory bowel diseases, highlighting the role of host-microbiome interactions in disease mechanisms (Franzosa et al., 2018). More recently, in the context of COVID-19, proteomic and metabolomic analyses revealed differential expression patterns correlated with disease severity. A predictive model was developed using 29 serum factors, including 22 proteins and 7 metabolites. This model demonstrated the potential for patient stratification, achieving an overall accuracy of 93.5% in the training set (Shen et al., 2020).

Collectively, these studies illustrate a broad landscape of applications for metabolomics that researchers can replicate across diverse diseases.

#### 3.4.2 Most frequent words and word dynamics

The top ten frequent author keywords were: metabolomics (3222 occurrences), biomarkers (1170), lipidomics (750), mass spectrometry (458), nuclear magnetic resonance (363), metabolites (316), proteomics (274), metabolome (249), and GC-MS (202). As expected, metabolomics and lipidomics were among the most frequently used words; however, it is interesting that proteomics also appeared in the list. Reviewing the keyword frequencies by disease, we found that Alzheimer’s disease (173), obesity (158), breast cancer (130), COVID-19 (127), and metabolic syndrome (109) were the highest.

Subsequently, we did other detailed analyses. The first was to elucidate the trending topics and their duration as trending. The second goal was to detect which diseases were the most studied in this approach and how they evolved.

**Figure 5:**
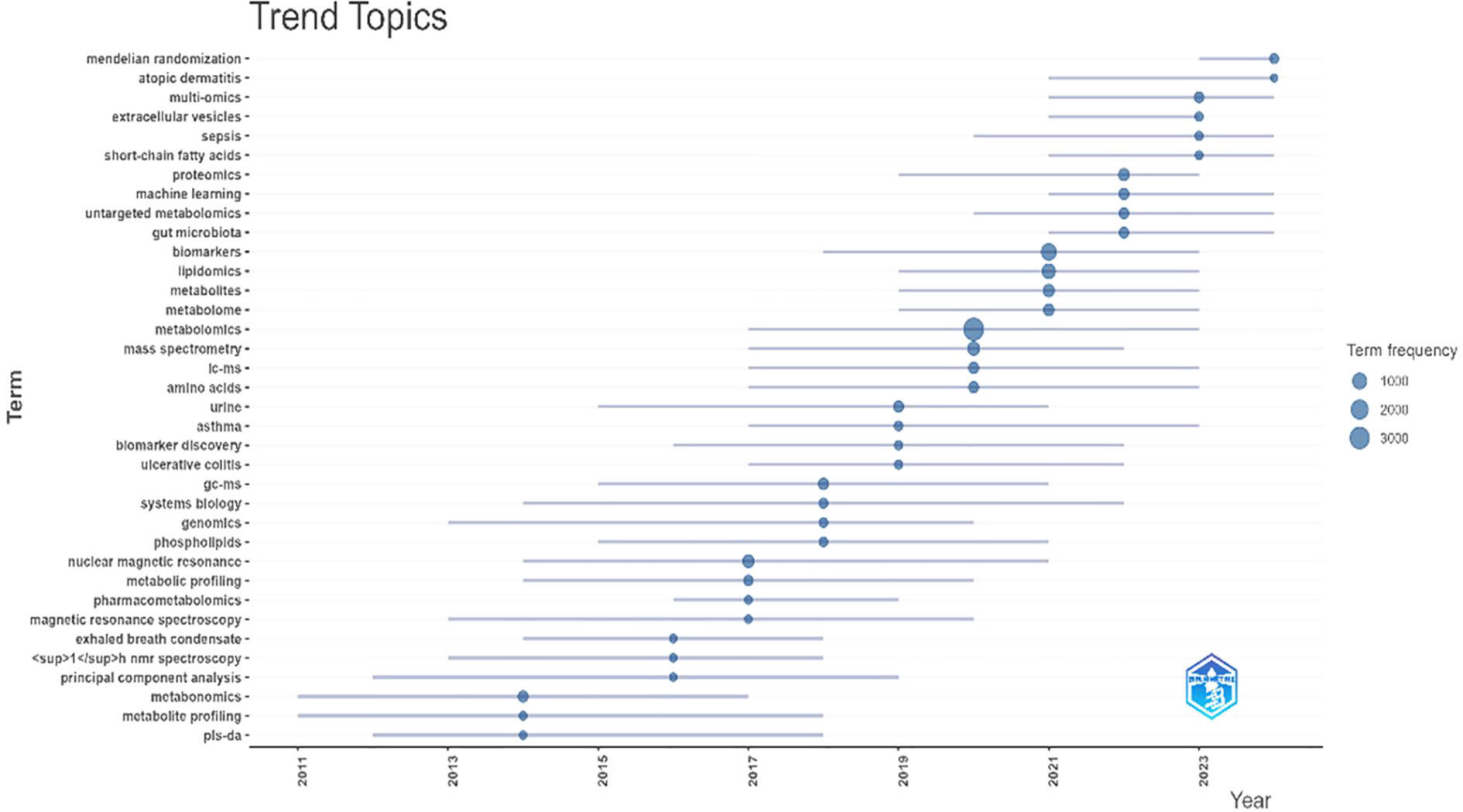
Trending topics. On the x-axis, we have the time span in years, and on the y-axis, the list of trending topics. The size of the circle indicates the frequency of the term, the position of the circle marks the year with the most publications on that topic, and the lines define how long that term has been present.

**Figure 5** shows trending topics from 2011 to 2024. We identified trending topics using Biblioshiny with the following settings: Author’s Keywords, a minimum frequency of 15 occurrences per year, a maximum of 4 words per year, and a timespan from 2011 to 2024. Then, we analyzed the appearance of trending topics through time. The size of each bubble reflects the frequency of the term, while the position of the bubble on the x-axis indicates the year with the highest frequency. We drew a timeline to indicate the duration of the term’s appearance over time, from its first to the third quartiles of the observed frequency.

As illustrated in **Figure 5**, there has been a notable surge in the adoption of methodologies such as Mendelian randomization and the utilization of machine learning algorithms. Additionally, diseases associated with lipidomics and metabolomics, such as asthma, atopic dermatitis and ulcerative colitis, are also included.

**Figure 6:**
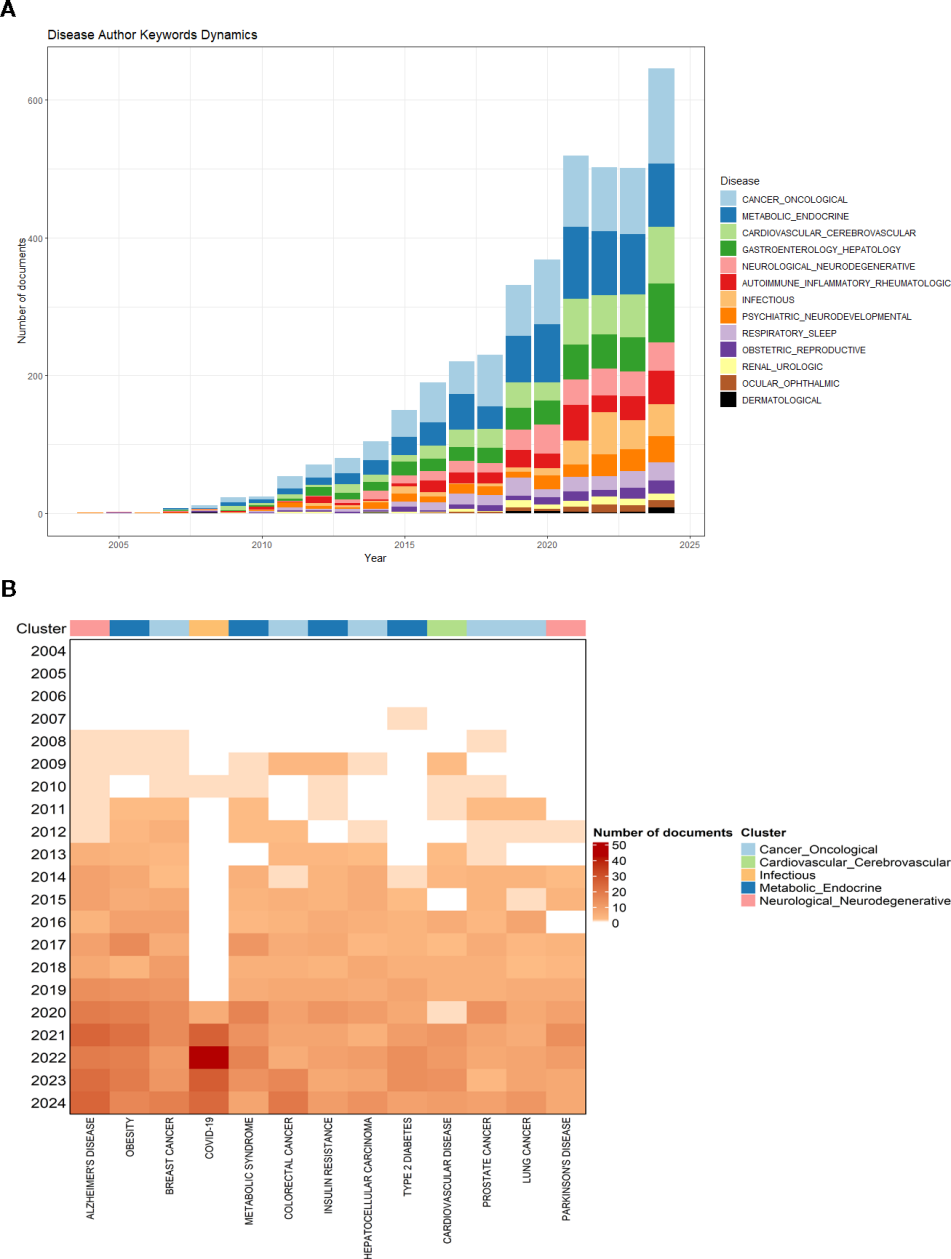
Disease author keywords dynamics. **(A)** Distribution of disease groups over time. The x-axis represents the time span in years, while the y-axis indicates the total number of documents. Each color corresponds to a disease category, and the height of each bar reflects the number of publications associated with each group per year. **(B)** Temporal dynamics of specific disease-related keywords. The x-axis displays the most frequently occurring disease terms (arranged from left to right according to overall frequency), while the y-axis represents the time span from earlier to more recent years (top to bottom). Color intensity indicates the number of documents, with darker shades representing higher publication frequency.

**Figure 6A** represents the most discussed disease groups and their interest over time. For example, the infectious appears to be more frequent from 2020 onwards, probably due to COVID-19. Then, cancer is also recurrent since there are numerous studies of breast cancer, colorectal cancer, and hepatocellular carcinoma, among others. Metabolic and endocrine diseases are also frequent, led by obesity, metabolic syndrome, and diabetes.

In **Figure 6B**, the temporal dynamics of the main disease-related terms identified using Biblioshiny are presented. From left to right, the terms are ordered according to their total frequency of occurrence. Rather than focusing on the absolute number of documents, given that this analysis is based solely on author keywords, this figure highlights changes in term concentration over time.

For instance, COVID-19 shows a marked increase, peaking around 2022, reflecting the global research response to the pandemic. Similarly, a growing concentration of publications in recent years can be observed for Alzheimer’s disease and colorectal cancer. In contrast, obesity, despite being one of the most commonly studied diseases associated with lipid-related research, appears to have reached a relative plateau, with increasing attention shifting towards other disease areas. Overall, both at the macroscopic (**Figure 6A**) and specific (**Figure 6B**) levels, an increase in disease-related research activity can be observed from 2020 onwards.

To further deepen the analysis, we selected the two most prominent clusters identified in **Figure 6B** (Cancer_Oncological and Metabolic_Endocrine) and conducted a targeted qualitative exploration of representative diseases within each cluster. This exploration was not intended to be a systematic review, but rather an illustrative analysis based on highly cited studies within our dataset, aimed at highlighting potential metabolic mechanisms associated with these diseases. It is important to note that identifying consistent metabolic patterns across diseases remains challenging, as results vary depending on study design, analytical platforms, and patient cohorts.

In the case of cancer, it is noteworthy that although all cancer types share the hallmark of uncontrolled cellular proliferation, each subtype exhibits distinct metabolic adaptations shaped by its tissue context and microenvironment.

In human colon carcinoma and colorectal cancer, metabolic studies have reported that intermediates of the TCA cycle and lipids are downregulated, whereas metabolites associated with the urea cycle, purines, pyrimidines, and amino acids are generally found at higher levels compared to normal colon mucosa (Denkert et al., 2008). Among these, specific amino acids such as methionine, cysteine, phenylalanine, and threonine have been reported as elevated. More recent studies further highlight the role of the human microbiome in colorectal cancer development, while coinciding in the observation that phenylalanine is significantly increased in intramucosal carcinomas (Weir et al., 2013; Yachida et al., 2019).

While similar analyses could be extended to other cancer types, identifying consistent metabolic patterns across diseases remains challenging due to biological heterogeneity and differences in study design. For example, in breast cancer, studies employing capillary electrophoresis-mass spectrometry-based saliva metabolomics have reported alterations in metabolites such as taurine and lysine compared to healthy controls (Sugimoto et al., 2010). Other approaches include proteogenomic analyses, which integrate mass spectrometry-based proteomics with next-generation DNA and RNA sequencing (Krug et al., 2020), as well as subtype-specific investigations, such as those focused on triple-negative breast cancer (Xiao et al., 2022) and estrogen receptor status (Alakwaa et al., 2018). These variations highlight how each analytical framework captures different aspects of tumor biology, making direct comparisons across studies inherently complex.

In the case of metabolic diseases, particularly type 2 diabetes and obesity, several studies report consistent associations between specific metabolite profiles and disease risk. For example, one study has identified branched-chain and aromatic amino-acids, including isoleucine, valine, tyrosine, and phenylalanine, as strongly associated with the future development of type 2 diabetes (Wang et al., 2011). Similarly, other studies have reported that serum metabolites such as hexoses, phenylalanine and specific diacyl-phosphatidylcholines are independently associated with increased risk of T2D, while metabolites such as glycine, sphingomyelins, and lysophosphatidylcholines are associated with decreased risk (Floegel et al., 2013). Notably, phenylalanine appears consistently across studies as a metabolite linked to disease risk.

In obesity, metabolomic profiles also reflect distinct alterations in lipid metabolism. Studies have shown increases in lysophosphatidylcholines, lipids associated with proinflammatory and proatherogenic conditions, and decreases in ether phospholipids, which are known to have antioxidant properties (Pietiläinen et al., 2007). Additional targeted metabolomics analyses have identified changes in amino acids such as glycine and glutamine, as well as alterations in specific phosphatidylcholine species (Oberbach et al., 2011), further highlighting the metabolic complexity of obesity.

Interestingly, while some metabolite patterns appear to differ between obesity and type 2 diabetes, these differences may reflect distinct stages of metabolic dysregulation rather than true contradictions. For instance, metabolites such as glycine have been reported at higher levels in certain obesity contexts, whereas they are consistently decreased in individuals at risk of T2D, highlighting the complexity and stage-dependent nature of metabolic signatures. Together, these findings underscore the need for validated biomarkers, standardized analytical pipelines, and open-access data repositories to facilitate the clinical translation of metabolomics and lipidomics in metabolic diseases.

**Supplementary Files 12**-**16** present the results from the analyses of the most frequent words, disease categories with inclusion and exclusion, their synonyms, and disease dynamics.

### 3.5 Conceptual structure

#### 3.5.1 Network approach: author keyword co-occurrence network

**Figure 7:**
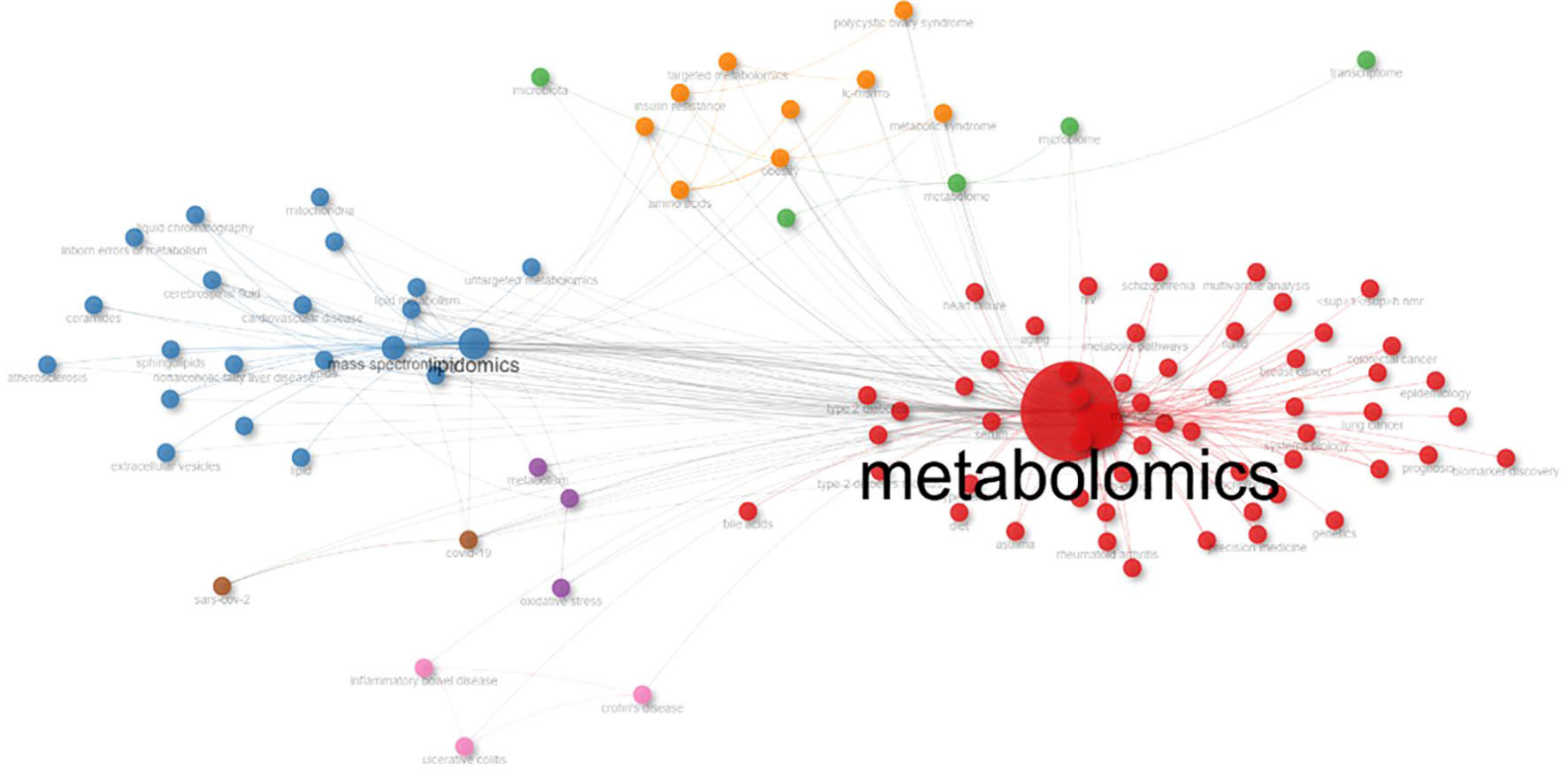
Author keyword co-occurrence network. We used the Louvain cluster algorithm and association strength normalization to make the network. A thicker line indicates a stronger relationship. The maximum number of nodes was 100, and the minimum number of edges was 10, but if more co-occurrences are found, the closer terms map to the center of the network. The keyword bubble size shows the frequency of use by academics. The color indicates different clusters and was based on the values of the features Betweenness, Closeness, and PageRank. The repulsion force was established as 0.1.

A co-occurrence network connects two author keywords with an edge when they appear together in a particular article, thereby revealing relationships between concepts. **Figure 7** presents the author keyword co-occurrence network, which we generated using the Louvain clustering algorithm with associated strength normalization.

Biblioshiny defined the clusters and their nodal positions based on the values of Betweenness, Closeness, and PageRank. The betweenness measure identifies the nodes that influence the flow of information within the system and characterizes the role of each cluster.

The analysis revealed several thematic clusters:

- Red cluster: strongly influenced by metabolomics (4119.735), biomarkers (351.519), nuclear magnetic resonance (3.324), and LC-MS (3.321).
- Blue cluster: characterized by lipidomics (158.901), mass spectrometry (65.707), and phospholipids (0.879).
- Green cluster: focused on metabolome (98.47), with links to microbiome (0.447) and gut microbiota (0.11).
- Purple cluster: centered on inflammation (0.4) and oxidative stress (0.009).
- Orange cluster: associated with amino acids (6.486), obesity (0.382), insulin resistance (0.155), and polycystic ovary syndrome (0.011).
- Brown cluster: linked to COVID-19 (0.617) and SARS-CoV-2 (0.0).
- Pink cluster: related to inflammatory bowel disease, Crohn’s disease, and ulcerative colitis (0.0).

#### 3.5.2 Network approach: thematic map

We treated the clusters of keywords obtained from the co-occurrence networks as themes. A thematic map evaluates these themes according to their position across two dimensions: centrality and density. Centrality measures the degree of interaction between a network and other networks, whereas density measures the internal strength of the network.

**Figure 8:**
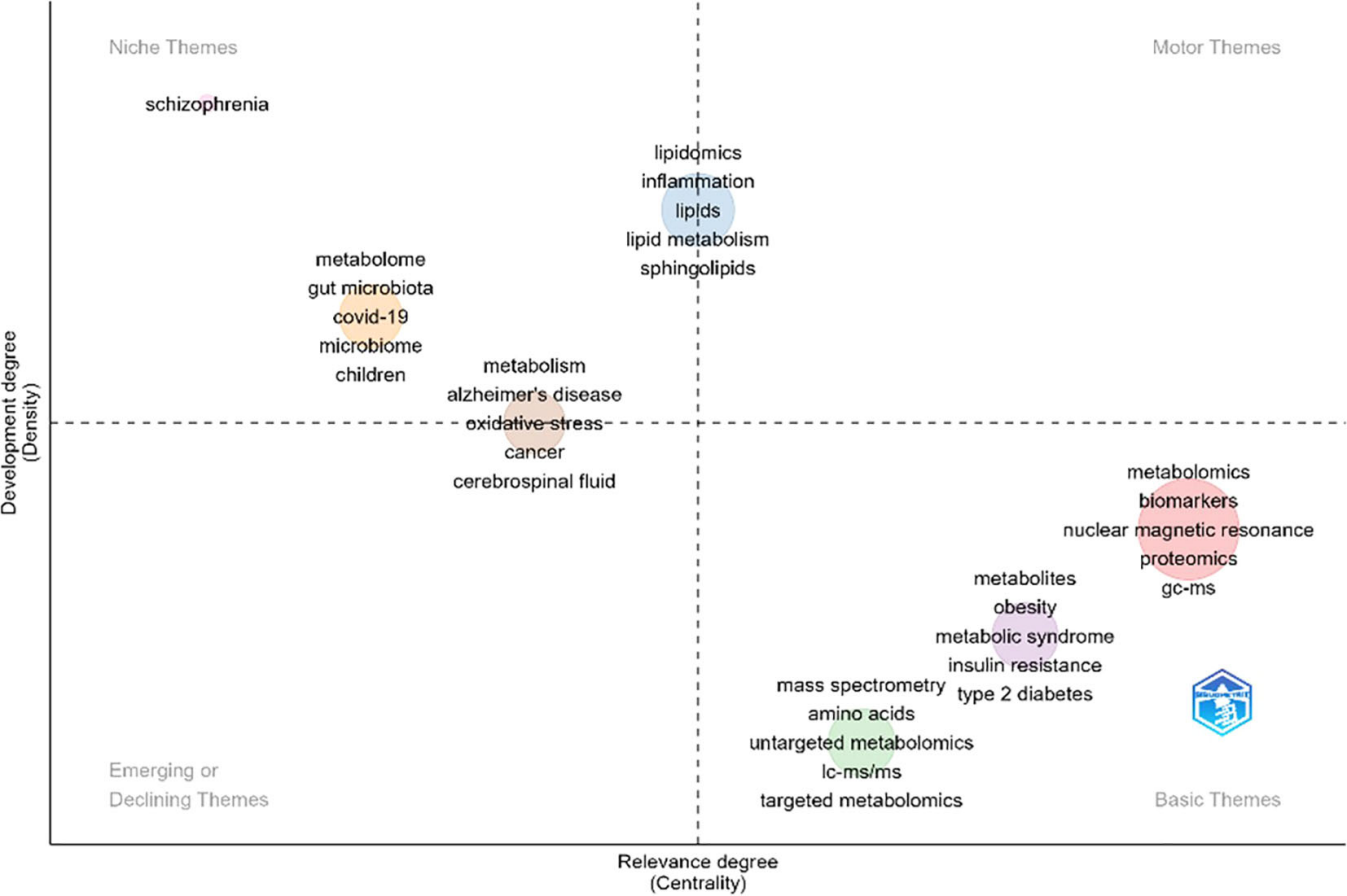
Thematic map. The number of words was defined as 1000 with a minimum cluster frequency (per thousand documents) of 5 using a Louvain clustering algorithm.

**Figure 8** represents the thematic map of our dataset, which contains seven clusters. The quadrants illustrate a different type of theme: the upper right quadrant displays the motor themes, the lower right quadrant shows the fundamental themes, the lower left quadrant contains the emerging or declining themes, and the upper left quadrant highlights the niche (specialized areas) themes.

Within the niche themes, which represent specialized areas with strong internal development but limited interconnection with the broader field, we identified the following topics:

- The relationship between the microbiome, gut microbiota, and SARS-CoV-2 has attracted significant attention during and following the COVID-19 pandemic. Although research in this area has advanced considerably, it does not yet constitute a central focus within the broader field.

Emerging evidence suggests that SARS-CoV-2 infection is associated with persistent alterations in gut microbial composition and metabolic function. For example, studies have reported that the gut microbiome of patients with COVID-19 exhibits a reduced capacity for the biosynthesis of short-chain fatty acids and essential amino acids such as L-isoleucine, with these alterations persisting even after disease resolution (Zhang et al., 2022).

These findings have been further supported by population-specific studies, including cohorts from Japan and Hong Kong, where integrated analyses have identified multiple correlations between COVID-19 associated microbial taxa (e.g., short-chain fatty acid producers) and gut-derived metabolites (Nagata et al., 2023). Together, these studies illustrate how microbiome-metabolite interactions constitute a specialized yet methodologically sophisticated research niche with growing translational relevance, consistent with its classification as a niche theme in the thematic map.

- The association between oxidative stress and cancer is a subject of considerable interest in oncology. Oxidative stress, an imbalance between the production of reactive oxygen species and the body’s antioxidant capacity, has been recognized as a key factor contributing to chronic diseases (Pizzino et al., 2017). Understanding this mechanism is crucial for developing preventive and therapeutic strategies in cancer and other conditions.
- Inflammation and sphingolipids appear grouped together, indicating an important area of investigation in immunometabolism. This thematic clustering reflects a growing body of research showing that inflammatory bowel diseases (IBD) such as Crohn’s disease and ulcerative colitis arise from disrupted interactions between the gut microbiome, host immune responses, and metabolic regulation.

For example, integrative multi-omic studies combining metagenomics and metabolomics have demonstrated that, compared with healthy individuals, patients with IBD exhibit enrichment of sphingolipids and bile acids alongside broad alterations in gut microbial composition. Moreover, metabolomic and metagenomic profiles have been shown to correlate strongly with fecal calprotectin levels, a clinical marker of intestinal inflammation, highlighting the close coupling between microbial metabolism and immune activity (Franzosa et al., 2018).

Within the fundamental themes, which encompass cross-cutting areas employed throughout the field, two groups stand out:

- The group of methodological approaches includes NMR, proteomics, GC-MS, LC-MS, and both targeted and untargeted metabolomics. These core techniques underpin the majority of studies in this field.
- The relationship with metabolic diseases, such as metabolic syndrome, insulin resistance, type 2 diabetes, and obesity, as well as amino acid metabolism. These problems are widely studied and remain central for metabolomics.

Numerous studies within our dataset address these metabolic disorders by linking circulating metabolites and lipids with clinical risk factors, inflammatory markers, and host-microbiome interactions. A representative example is a recent study from our group, which identified plasma lipidomic signatures associated with gut microbiota alterations in children and adolescents with type 2 diabetes mellitus and metabolic syndrome. This work demonstrated coordinated changes in phosphocholines, sphingolipids, triglycerides, and ceramides that correlated with obesity, insulin resistance, and specific microbial taxa, illustrating how lipidomics can integrate metabolic, inflammatory, and microbial dimensions of disease pathophysiology (Mora-Godínez et al., 2025).

We set the number of words to 1000 and the minimum cluster frequency to 5 to capture both central and peripheral topics. While this setting may introduce minor variability in cluster assignment across different runs due to the stochastic nature of the Louvain algorithm, the main thematic structures (e.g., metabolomics, lipidomics, biomarkers) remained robust, supporting the reliability of our analysis.

#### 3.5.3 Network approach: thematic evolution map

**Figure 9:**
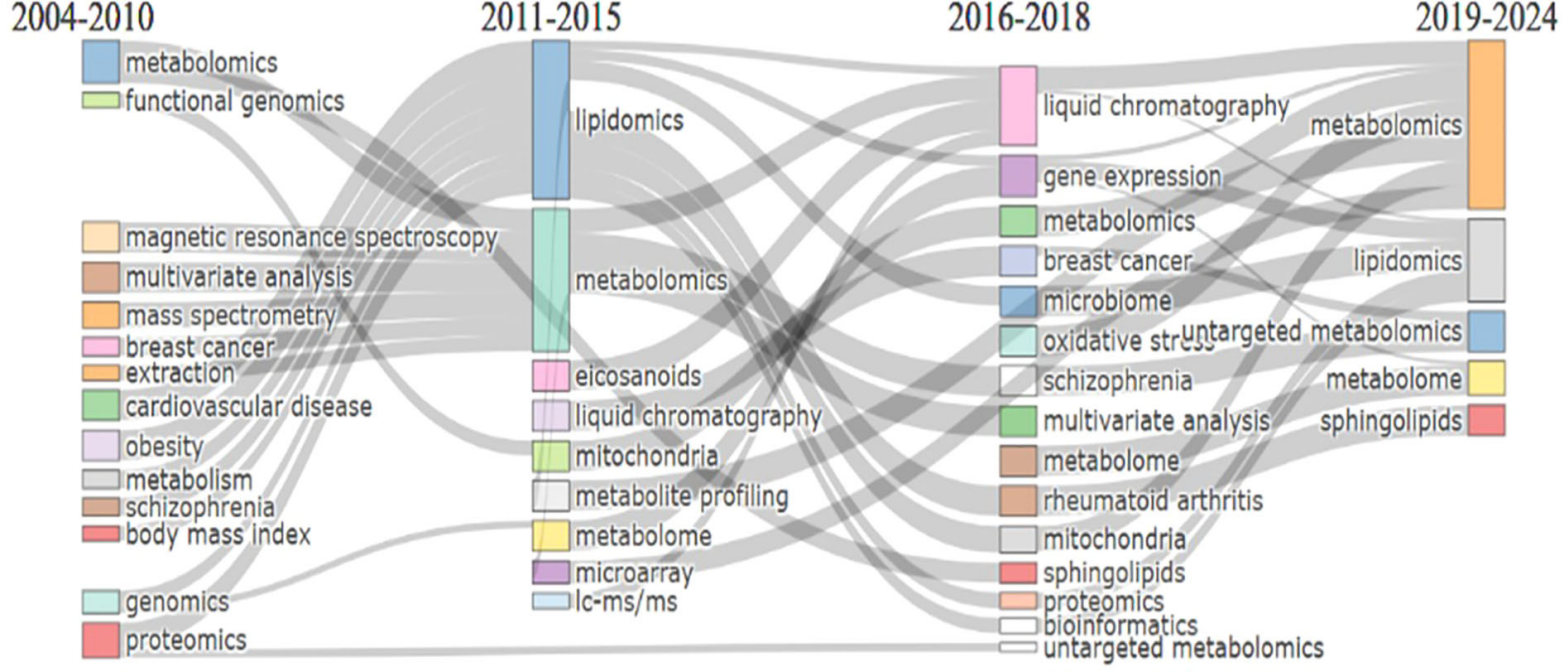
Thematic evolution map. The analysis was based on the 1,000 most frequent author keywords (minimum cluster frequency of 5 per thousand documents), identified through the Walktrap clustering algorithm.

**Figure 9** presents a thematic evolution map that illustrates the evolution of topics across different time slices. We defined these slices according to the number of publications reported in **Figure 2**.

Regarding the methodologies, from 2004 to 2010, research emphasized classical analytical techniques such as magnetic resonance spectroscopy and mass spectroscopy. During 2011-2015, more specific methods, such as LC-MS, consolidated the technical foundation of the field. From 2016 to 2024, these methods persisted mainly as cross-cutting tools, while broader conceptual approaches gained prominence.

In the early stages (2004-2010), disease prevalence centered on obesity, cancer, and cardiovascular disease, later expanding to more specific or immunologically relevant conditions such as rheumatoid arthritis and its link to oxidative stress (2016-2019). By 2019-2024, the emphasis shifted toward a more integrative perspective. Although disease-specific research remained central, disease terms became less prominent among the most frequent or central keywords. This trend reflects a conceptual transition in which lipidomics, and metabolomics methodologies increasingly serve as cross-cutting frameworks applied to diverse pathological contexts, rather than isolated disease domains.

Notable examples of this integrative perspective include longitudinal multi-omic studies that combine proteomics, metabolomics, transcriptomics, and immunological profiling to investigate disease trajectories. For instance, one study applied a longitudinal multi-omics framework to predict the onset of post-acute sequelae of COVID-19, following patients from diagnosis through acute disease and subsequent convalescence (Su et al., 2022). This design illustrates how metabolomics and lipidomics can be embedded within hypothesis-driven, time-resolved analytical frameworks that link molecular variation to clinical outcomes.

Another representative example is the use of integrated multi-omics approaches to elucidate interactions between the microbiome, disease mechanisms, and therapeutic responses in ulcerative colitis (Mills et al., 2022). In this study, metaproteomics, metapeptidomics, metabolomics, and host serum proteomics were jointly analyzed, highlighting how metabolic-derived signals contribute to host-microbe interactions. In such frameworks, lipidomics could provide complementary insights into membrane composition, inflammatory signaling, and metabolic regulation that cannot be fully captured by other omics layers alone.

Importantly, these integrative research models rely on mass spectrometry-based analytical platforms, which in practice are predominantly LC-MS, enabling the coherent integration of metabolites, lipids, and related molecular features across omic layers. These examples illustrate how lipidomics and metabolomics operate as cross-cutting frameworks that bridge molecular, computational, and clinical dimensions, consistent with the thematic convergence observed in the 2019–2024 period of the evolution map. The map also highlights that metabolomics remained the dominant field, while lipidomics consolidated as an independent and increasingly significant subfield.

### 3.6 Cross-database validation of results

To assess the robustness of the Scopus-based findings, equivalent searches were conducted in WoSCC and PubMed, yielding 7,333 and 5,096 publications, respectively, within the defined time frame and inclusion criteria. While absolute publications counts varied due to differences in database coverage, the temporal trends of annual publications were highly consistent across the three databases, with coefficients of determination (R^2^) of 0.992 (Scopus vs WoSCC) and 0.995 (Scopus vs PubMed).

**Figure 10:**
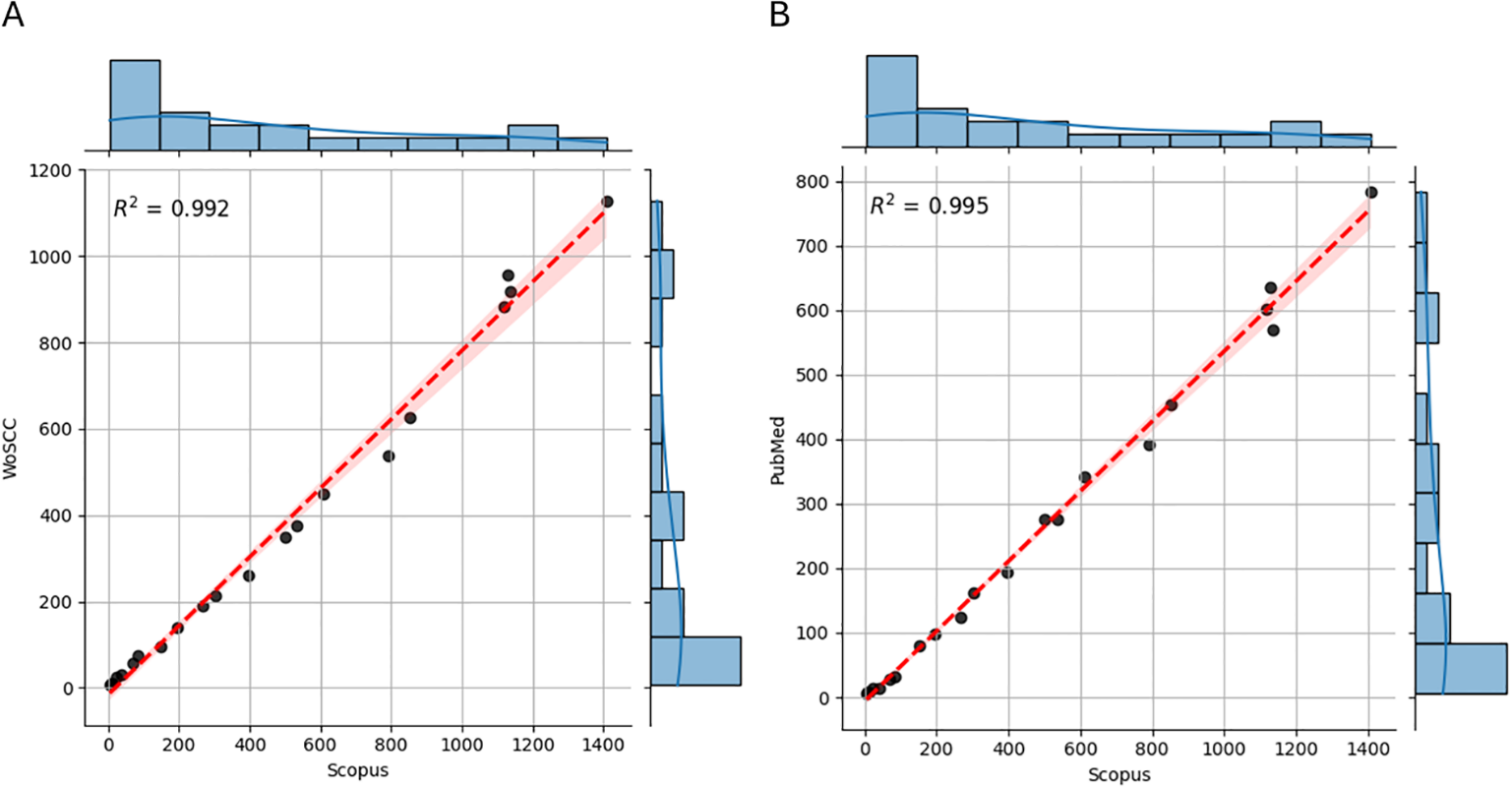
Cross-database validation of annual publications. The joint plot combines central scatterplots showing the distribution of publications from 2004 to 2024 between Scopus and the other databases. Marginal Histograms on the top and right axes depict the individual distribution of each variable. The red line represents the linear regression line of best fit, and the shaded ban around it indicates the confidence interval. Each point represents a year of publications, and these are consistently matched between databases. **(A)** Scopus vs WoSCC. **(B)** Scopus vs WoSCC.

As illustrated in **Figure 10**, the joint plot combines central scatterplots showing the distribution of publications from 2004 to 2024 between Scopus and the other databases. Marginal histograms on the top and right axes depict the individual distribution of each variable. The red line represents the linear regression line of best fit, and the shaded ban around it indicates the confidence interval. The high R^2^ values further validate the remarkably strong correlation among databases.

The histograms reveal right-skewed distributions, reflecting a predominance of low publication counts in the early years, followed by a marked increase in recent years. Annual growth rates were comparable across sources – 32.58% in Scopus, 29.93% in WoSCC, ad 27.58% in PubMed. Similar consistency was observed in document age average, international co-authorship, and co-authors per document, with each fitted model showing an adjusted R^2^ of 0.99.

Regarding journals, the most productive titles remained consistent across databases, including Scientific Reports, Metabolomics, PLoS ONE, and the Journal of Proteome Research, among others. Likewise, the United States and China consistently ranked first and second in publication volume, followed by major European contributors, while Nordic countries showed the highest international collaboration ratios.

Trending topics also showed strong reproducibility, with recurring themes such as microbiome, Mendelian randomization, machine learning, and multi-omics integration. Minor variations were observed in keyword frequencies, mainly due to database-specific indexing policies and journal coverage. However, no substantive differences emerged in the overall trajectory, thematic priorities, or geographical distribution of research.

These results confirm that the observed trends, key contributors, and thematic structures are not artifacts of a single database but represent stable and reproducible patterns across major bibliographic platforms.

**Supplementary Files 17**, **18** provide the search queries, tables, and figures corresponding to the analyses conducted for WoSCC and PubMed, respectively.

## 4 Discussion

### 4.1 Performance analysis

The annual growth rate of 32.58% confirms the rapid rise of lipidomics and metabolomics as diagnostic tools. The acceleration observed over the last decade reflects both the consolidation of technologies such as LC-MS and NMR and the growing clinical interest in biomarkers. Polynomial adjustment and projections for 2025 suggest continued expansion of the field. However, it remains essential to evaluate the quality of publications, not only their quantity. We emphasize that these projections represent an exercise based on historical trends and do not account for unforeseen events, such as a decline in interest in the field or the emergence of another pandemic like COVID-19. Therefore, these predictions should be interpreted with caution, as they depend on the current conditions of research activity.

From the analysis of journals, we observed a clear concentration of publications in Scientific Reports and PLoS ONE, highlighting the importance of multidisciplinary open-access outlets. The presence of clinical and analytical chemistry journals further demonstrates the interdisciplinary nature of the field. Moreover, the diversity of bibliometric indicators reinforces that the impact of this domain is both methodological and biomedical.

The United States and China dominate in publication volume, followed by the United Kingdom, Germany, Italy, and Spain that represent the leading contributors in Europe, and when combined, they surpass each of the two global leaders individually. International collaborations of 31.38% and an average of 9.91 co-authors per document highlight the inherently multidisciplinary and global nature of lipidomics and metabolomics research. Notably, the Nordic countries – Finland, Sweden, and Denmark – exhibit exceptionally high collaboration rates, offering a potential model for fostering international partnerships.

The pronounced geographic concentration of lipidomics and metabolomics research in the United States, China, and Europe reflects not only scientific leadership, but also structural disparities in access to advanced analytical infrastructure, specialized training, and sustained research funding. High-throughput platforms such as LC-MS/MS and NMR require substantial capital investment, standardized workflows, and highly trained personnel, conditions that remain unevenly distributed across regions.

Evidence from international surveys supports this interpretation. A recent global assessment conducted by the International Federation of Clinical Chemistry and Laboratory Medicine reported that although clinical interest in metabolomics is growing worldwide, major barriers to implementation include technical complexity, lack of assay standardization, high costs, limited availability of commercial tests, and insufficiently trained staff (Fux et al., 2024). Notably, more than half of respondents reported only basic or limited experience in metabolomics, underscoring a global skills gap that likely contributes to regional disparities in research output. There is also a disparity in the use of LC-MS/MS (83%) compared to NMR (9%) among those who use metabolomics.

These constraints are further compounded by challenges in scaling metabolomics to large cohorts, as highlighted by recent methodological analyses emphasizing the need for standardized operating procedures, interoperable data infrastructures, and dedicated training programs to enable routine, large-scale application across disciplines (Hajjar et al., 2023). Together, these factors suggest that the observed geographic concentration reflects both real infrastructure limitations and, to some extent, database indexing biases that favor well-resourced research environments.

Addressing these inequities will be essential for ensuring that lipidomics and metabolomics contribute meaningfully to global health, particularly in low- and middle-income countries where metabolic diseases impose a growing burden. Investment in shared infrastructures, open-access analytical resources, capacity-building initiatives, and international collaborations will be critical to democratizing access to omics-based diagnostic innovation.

### 4.2 Science mapping

Using a network approach, we identified the main thematic areas and trends in lipidomics and metabolomics research. In summary, the basic topics across the field remain methodological techniques (LC-MS, GC-MS, NMR) and metabolic diseases (diabetes, obesity, metabolic syndrome). Among the niche themes, we observed microbiota-COVID-19, inflammation and sphingolipids, oxidative stress, and cancer – topics of relevance that remain peripheral to the core of the field.

Beyond describing dominant themes, the observed patterns reflect structural and translational realities of the field. The prominence of metabolic diseases likely reflects both their high global disease burden (Zhang et al., 2024) and their suitability for metabolomic interrogation (Park et al., 2015), as these conditions involve systemic metabolic dysregulation that is readily captured by lipid and metabolite profiling. In contrast, diseases with more localized or heterogeneous pathophysiology appear less frequently, highlighting and unmet need for methodological adaptation and clinically scalable workflows.

We also identified emerging themes, including machine learning, Mendelian randomization, and multi-omics integration. The transition from disease-specific approaches to broader cross-cutting frameworks suggests both the maturity of the field and its potential for clinical translation. These advances reflect the increasing incorporation of computational and integrative methodologies that allow researchers to extract deeper biological insights from complex metabolic data.

#### 4.2.1 Machine learning

Machine learning (ML) is a subdiscipline of artificial intelligence that refers to algorithms capable of learning from data without explicit programming. In biomedical research, ML has become a key analytical framework for handling large-scale and high-dimensional datasets typical of lipidomics and metabolomics studies. ML methods are broadly classified into supervised and unsupervised learning, depending on whether the outcome variable is known during model training (Zadeh Shirazi et al., 2024).

Supervised learning uses labeled datasets to train predictive models capable of classifying or quantifying new observations. Typical applications in metabolomics include distinguishing patients with and without disease based on lipid and metabolite profiles, or predicting a metabolite concentration using regression-based approaches. For instance, researchers developed support vector machine (SVM) models using lipidomic and glycomic data to distinguish between healthy individuals, non-alcoholic fatty liver, and non-alcoholic steatohepatitis patients with high diagnostic performance, with sensitivities and specificities above 90% and areas under the ROC curve approaching 0.9 (Perakakis et al., 2019). Common algorithms include SVM, k-nearest neighbors, and decision trees, as well as ensemble models that combine multiple learners, such as random forests, bagging, and boosting (Galal et al., 2022). In applying these models, researchers must balance the trade-offs between model complexity and prediction error, and between accuracy and interpretability – a key consideration for clinical translation, as interpretability often incurs only a limited penalty in predictive performance (Johansson et al., 2011).

Unsupervised learning identifies patterns or subgroups within unlabeled data, often used to detect disease subtypes or molecular signatures (Lehmann et al., 2011). Clustering techniques, such as k-means, hierarchical clustering, and principal component analysis (Eckhardt et al., 2023) are commonly employed to reveal hidden structures in metabolomic datasets (Gal et al., 2020). These models can stratify patients according to metabolic similarity, generating hypotheses for precision medicine.

Deep learning, a subfield of ML, relies on multi-layered neural networks capable of processing complex, nonlinear relationships in large datasets. These architectures excel at pattern recognition and high-dimensional feature extraction but often lack interpretability compared to traditional ML approaches – a limitation that remains a critical issue for clinical metabolomics (Pomyen et al., 2020). As deep learning models become increasingly complex, the field is moving toward explainable artificial intelligence (XAI) approaches that prioritize transparency, justification, and control over model decisions – key requirements for trustworthy clinical applications (Sadeghi et al., 2024).

#### 4.2.2 Mendelian randomization

We highlight the role of Mendelian randomization given its prominence as an emerging theme. This methodological framework enables the investigation of the causal effects of modifiable exposures (i.e., potential risk factors) such as excessive alcohol consumption and tobacco use on health, social, and economic outcomes. Mendelian randomization rests on the principle that genetic variants arise during meiosis and are therefore not influenced by lifestyle, environment, or personal decisions (Richmond and Davey Smith, 2022).

To illustrate, the single-nucleotide polymorphism (SNP) rs429358 determines whether a cytosine (C) or thymine (T) allele is present at its locus within the APOE gene. The SNP rs7412 likewise carries either a C or a T allele at its position. Considered together, these two polymorphisms define the three APOE isoforms: ϵ2 (T at rs429358 and T at rs7412), ϵ3 (T at rs429358 and C at rs7412), and ϵ4 (C at rs429358 and C at rs7412). The ϵ4 isoform is associated with increased risk of Alzheimer’s disease, whereas ϵ2 has been linked to greater longevity. Mechanistically, APOE plays a central role in lipid metabolism, mediating the clearance of triglyceride-rich lipoproteins (Corder et al., 1993; Seripa et al., 2011). APOE and its dual connection with Alzheimer’s disease and lipid metabolism exemplify how genetics converge with metabolomics.

These genetic variants can serve as instruments to evaluate causal relationships. For instance, if a genetic variant is associated with a risk factor (e.g., elevated LDL cholesterol) and a disease (e.g., cardiovascular disease), then we can infer that the risk factor may have a causal effect on the disease. This approach permits researchers to test causality in contexts where clinical trials would be ethically or logistically infeasible. For example, it would be unethical to artificially elevate cholesterol levels in humans to observe whether they develop cardiovascular disease. However, if a genetic variant that increases cholesterol also increases disease risk, the causal hypothesis is reinforced with greater clarity (Larsson et al., 2023).

For Mendelian randomization to be valid, its fundamental assumptions must hold:

1. The SNP must show a strong association with exposure (relevance).
2. The SNP must be independent of potential confounders (independence).
3. The effect of SNP on the outcome must operate exclusively through the exposure (exclusion restriction) (Richmond and Davey Smith, 2022).

#### 4.2.3 Multi-omics integration

Metabolomics also represents a close counterpart to the genome, the transcriptome, and the proteome; together, these four ‘omes’ constitute the fundamental building blocks of systems biology (Wishart et al., 2007). It is believed that recent technological advances in these omics, combined with wearable monitoring, will enable precision health approaches that rely on the ability to assess disease risk at the individual level, detect early preclinical conditions, and initiate preventive strategies (Schüssler-Fiorenza Rose et al., 2019). The integration of these multi-dimensional data provides a rationale for therapeutically targeting microbe-host interactions in disease (Yan et al., 2022). An illustrative example in the field of metabolic disorders is the international effort to integrate multi-omics data on childhood obesity and metabolic dysfunction, which has uncovered biological pathways and prenatal determinants of disease risk (Stratakis et al., 2025).

Taken together, our network analyses and thematic mapping highlight how the field has evolved from a focus on methodological development and specific diseases to broader frameworks that integrate genetics, systems biology, and computational methods. This transition highlights the advancing maturity of lipidomics and metabolomics, where Mendelian randomization and multi-omics integration in conjunction with machine learning are poised to enhance causal inference and expedite clinical translation.

These findings demonstrate that bibliometrics not only describes scientific production but also reveals bridges between techniques and underlying mechanisms.

### 4.3 Strengths and limitations

Among the main strengths of this study is its integrative and comparative scope. Recent bibliometric studies have successfully characterized the evolution of lipidomics as an independent research field. For example, Li et al., 2025. provided a comprehensive single-omics overview of lipidomics using WoSCC data, emphasizing publication growth, co-citation structures, and emerging thematic hotspots. While such analyses are essential for understanding disciplinary development, they inherently examine lipidomics in isolation.

In contrast, this research adopts an integrated lipidomics-metabolomics framework, allowing for the identification of cross-omic convergence patterns that are not readily observable in single-omics bibliometric analyses. By jointly analyzing both omics domains, we reveal shared disease clusters, overlapping analytical platforms, and computational approaches, such as machine learning and Mendelian randomization, that function as conceptual and methodological bridges between fields. Moreover, the incorporation of text-mining strategies focused on disease categories highlights translational trajectories across multiple pathologies, rather than within a single omics context.

This integrative perspective extends beyond mapping disciplinary growth and instead captures how lipidomics and metabolomics co-evolve toward diagnostic-oriented and systems-level research, providing insights that complement, rather than replicate, prior single-omics bibliometric studies.

Additional strengths include the study’s broad temporal coverage (2004-2024) and its representative sample of more than 9,600 articles. The combined use of Bibliometrix, Scimago Graphica, R scripts, and OpenRefine ensured data cleanliness and provided multiple analytical perspectives. Similarly, the integration of classic bibliometric indicators (h, g, m) with co-occurrence networks and thematic evolution maps enhanced the robustness of our results.

Methodological rigor was also prioritized. Only articles with complete metadata were considered, and author’s keywords were harmonized to minimize inconsistencies and allow reliable interpretations of scientific production. Furthermore, we carefully reviewed the results generated by Biblioshiny and complemented them with custom code developed in-house by the research team. This approach enabled us to refine metrics of authors, affiliations, and countries, producing evaluations that in some cases surpassed those provided by Bibliometrix, for example, the total number of citations by corresponding authors. Beyond quantitative metrics, we also examined the content of publications, summarizing the main research interests of authors and analyzing the dynamics of key terms such as databases and disease groups.

Importantly, this research also incorporated a multi-database validation component using WoSCC and PubMed, which confirmed the stability of the main patterns observed in Scopus. This cross-comparison supports the reliability of the findings and offers a stronger foundation for future basic and translational research in lipidomics and metabolomics applied to disease diagnosis.

However, some limitations must be acknowledged. Although cross-database validation was performed, Scopus remained the primary and most detailed data source, which may have excluded studies indexed exclusively in Web of Science or PubMed. While similar analyses could theoretically be conducted using other databases, the integration of multiple bibliographic sources is technically challenging and often unreliable due to differences in indexing structures, metadata formats, and citation metrics (Öztürk et al., 2024).

Scopus was selected because previous comparative studies have demonstrated its extensive coverage, particularly in biomedical research and computationally oriented fields, as well as its comprehensive citation tracking and author profiling capabilities (Pranckutė, 2021). Nevertheless, like other major databases, Scopus is subject to biases related to language, regional representation, and disciplinary coverage. These limitations were considered when interpreting the results and underscore the need for cautious generalization.

Second, although we standardized terms using OpenRefine, some bias may persist due to manual harmonization. Rare cases where keyword merging was necessary may not have been detected. For example, certain influential papers, such as “MetaboAnalyst 5.0: narrowing the gap between raw spectra and functional insights” (Pang et al., 2021), were not retrieved by our search despite their high citation impact. MetaboAnalyst, a web-based platform for metabolomics data analysis and multi-omics integration, illustrates the importance of complementing bibliometric approaches with specialized resources in the field. Conversely, a few retrieved articles may represent false positives, such as “Standardization of sample collection, isolation, and analysis methods in extracellular vesicle research” (Witwer et al., 2013). We believe, however, that such cases are minimal given the stringent and refined search strategy applied.

Finally, an important limitation of this study is that Scopus does not allow the export of cited references, which restricted the scope of citation-based analysis. As a result, while total citation counts were analyzed to assess overall impact, it was not possible to evaluate local citation patterns within the lipidomics and metabolomics field, nor to construct co-citation networks, historiographies, or citation clusters.

These analyses are particularly relevant for characterizing the intellectual structure of a field, as they allow identification of foundational works, citation lineages, and groups of documents that are frequently cited together over time. Consequently, the present study emphasizes research productivity, thematic evolution, and conceptual trends rather than fine-grained citation interdependencies.

Importantly, this limitation does not affect the robustness of the reported performance indicators or thematic mappings, but it does highlight the need for complementary studies using databases that support cited-reference export to further explore the intellectual genealogy of lipidomics and metabolomics human disease research.

### 4.4 Implications and future directions

The field is moving toward multi-omics integration, the application of artificial intelligence and machine learning, and the exploration of the microbiota-metabolism-brain axis. These advances are paving the way for the clinical translation of lipidomics and metabolomics, supported by validated biomarkers and open-access repositories that enhance reproducibility and transparency.

Looking ahead, the discipline’s progress will depend on several interrelated factors. Continued advances in data standardization will be essential to ensure reproducibility and interoperability across studies, enabling meaningful cross-platform comparisons. Equally important will be the establishment of large, international cohorts that can generate robust and generalizable findings across diverse populations. Finally, sustained interdisciplinary collaboration – particularly among clinicians, bioinformaticians, and system biologists – will be crucial to bridge methodological innovation with biomedical application. Together, these efforts will accelerate the transition of lipidomics and metabolomics from research-driven domains to actionable tools in precision health and clinical diagnostics.

## Data Availability

The data presented in the study are deposited in the Zenodo repository, DOI/accession number: https://doi.org/10.5281/zenodo.20089065.

## Supplementary material

Supplementary File 1 Table 1.DOCX (Supplemntary_Material_MainMethods.docx) Detailed description of the search query.

Supplementary File 2 Data Sheet 1. CSV (Scopus_exported_refine_values.csv) Refined search values from the Scopus database.

Supplementary File 3 Table 2.XLSX (Scopus_lipmet_all.xlsx) BibTeX file with the original dataset of 9728 articles from Scopus converted to XLSX for review.

Supplementary File 4 Table 3.XLSX (Bibliometrix-Export-File-2025-02-15.xlsx) Excel file containing 9628 documents to be reviewed and recognized by Bibliometrix.

Supplementary File 5 Image 1.JPEG (Completeness.jpg) Biblioshiny report indicates the completeness of metadata.

Supplementary File 6 Data Sheet 2.CSV (Key_Collision_Clusters.csv) Excel file containing the Key Collision Clusters reported by OpenRefine.

Supplementary File 7 Table 4.XLSX (NN_Clusters) Excel file containing the Nearest Neighbors Clusters accepted and reported by OpenRefine.

Supplementary File 8 Table 5.XLSX (Harmonized_Bibliometrix-Export-File-2025-02-15.xlsx.xlsx) Harmonized dataset with updated keywords processed through OpenRefine.

Supplementary File 9 Data Sheet 3.CSV (Annual_Production_bibliometrix_2025-02-17.csv) Annual production data used for analysis.

Supplementary File 10 Data Sheet 4.CSV (countries results.csv) Results by country detailing their contributions to the dataset.

Supplementary File 11 Data Sheet 5.CSV (Collaboration_WorldMap.csv) Collaboration data between countries in the dataset.

Supplementary File 12 Table 6.XSLX (Most_Frequent_Words_bibliometrix_2025-06-19.xlsx) List of the most frequent words found in the dataset.

Supplementary File 13 Table 7.XSLX (Disease_Categories_Inclusion-Exclusion.xlsx) Most frequent disease keywords with its category.

Supplementary File 14 Data Sheet 6.CSV (Disease_Categories_Synonyms.csv) Synonym list for disease-related terms used in harmonization.

Supplementary File 15 Data Sheet 7.CSV (Word_Dynamics_bibliometrix_2025-08-16.csv) Dynamics of disease-related records within the dataset.

Supplementary File 16 Table 8.XLSX (Word_DiseaseSpecificDynamics_bibliometrix_2026-04-17.xlsx) Dynamic of specific diseases-related records within the dataset.

Supplementary File 17 Table 9.DOCX (Supplementary_Material_WoSCC.docx) Validation analysis from WoSCC.

Supplementary File 18 Table 10.DOCX (Supplemntary_Material_PubMed.docx) Validation analysis from PubMed.
