# Supplementary material for: A bibliometric and text-mining analysis of lipidomics and metabolomics in human disease
Source: Front Physiol. 2026 May 19;17:1727465. doi: 10.3389/fphys.2026.1727465 (PMC13225983; doi:10.3389/fphys.2026.1727465)
Supplement: Supplementary File 1 — Table 1.DOCX (Supplemntary_Material_MainMethods.docx) Detailed description of the search query. [file Table1.docx]

Supplementary Material – Main Methodology

# Search Strategy

The Scopus database was queried on February 8, 2025, using the following search strategy:

TITLE-ABS-KEY ( ( lipidome OR lipidomics OR metabolome OR metabolomics ) AND ( affliction OR ailment OR condition OR disease OR disorder OR illness OR infection OR malady OR morbus OR pathology OR syndrome ) AND ( analysis OR assessment OR detection OR diagnosis OR determination OR evaluation OR examination OR identification OR investigation OR screening ) AND ( human* OR patient* OR clinical OR medicine OR biomedical OR healthcare OR hospital ) AND NOT ( "in vivo" OR nonhuman OR animal OR mice OR mouse OR rat* OR rodent OR canine OR pig OR swine OR "animal model" ) AND NOT ( plant* OR vegetation OR crop* OR agriculture OR agronomy OR botany OR phytology OR phytochemical OR "plant-derived" OR "botanical extract" OR "phytotherapy" OR "plant model" ) ) AND PUBYEAR > 2003 AND PUBYEAR < 2025 AND ( LIMIT-TO ( DOCTYPE , "ar" ) ) AND ( EXCLUDE ( SUBJAREA , "BUSI" ) OR EXCLUDE ( SUBJAREA , "ECON" ) OR EXCLUDE ( SUBJAREA , "ARTS" ) OR EXCLUDE ( SUBJAREA , "EART" ) OR EXCLUDE ( SUBJAREA , "ENER" ) OR EXCLUDE ( SUBJAREA , "VETE" ) OR EXCLUDE ( SUBJAREA , "PHYS" ) OR EXCLUDE ( SUBJAREA , "AGRI" ) OR EXCLUDE ( SUBJAREA , "PLANT" ) ) AND ( LIMIT-TO ( LANGUAGE , "English" ) ).

The strategy was designed to capture literature related to lipidomics and metabolomics in human biomedical research while excluding studies based on animal or plant models, as well as publications from non-biomedical subject areas. Only original research articles written in English and indexed up to 2024 were included.

The search results were exported in BibTeX (.bib) and comma-separated values (.csv) formats. BibTeX files were used for subsequent bibliometric analyses, while CSV files were employed for data verification and access. Due to Scopus export limitations (maximum 2,000 records per download), results were retrieved in multiple blocks and subsequently integrated using Texmaker (Brachet, 2023).

The final curated dataset served as the basis for all downstream analyses described in the main manuscript.

# Data Filtering

Data filtering was conducted using the Biblioshiny Shiny application from the open-source Bibliometrix package (Aria and Cuccurullo, 2017; Derviş, 2020). Bibliometrix is widely used in bibliometric research because it enables data import from multiple bibliographic databases, supports science mapping analyses, and provides performance indicators as the source, author, and document levels within conceptual intellectual, and social knowledge structures (Linnenluecke et al., 2020; Moral-Muñoz et al., 2020).

From the initial collection of 9,728 articles retrieved from Scopus, 9,628 records contained complete bibliographic metadata and were retained for further analysis. The filtered dataset was exported for manual inspection, and a report of metadata completeness generated by Biblioshiny was archived to documents the filtering process.

# Data Harmonization

Bibliometric analyses require a preliminary data preparation phase because of potential data inaccuracies. This data-cleaning stage plays a crucial role in ensuring the credibility and reliability of the findings. In our collection, we analyzed the authors’ keywords, since these terms best capture the essence of an article’s subject matter. For instance, when addressing Cushing’s syndrome, an endocrine disorder marked by elevated cortisol production, researchers might employ keywords such as “Cushing’s syndrome”, “Cushing syndrome”, or “Cushings syndrome”. Ideally, we unified these terms to reflect a consensus on the expression of the same disease across various articles.

To automate facet cleaning by clustering terms, we employed the Java-based OpenRefine 3.8.2, an open-access tool for managing and transforming complex data (Ham, 2013; CS&S, 2024). The procedure involved three steps: dividing multi-valued cells, extracting text facets, and amalgamating multi-valued cells.

We applied the Key Collision method and the Fingerprint function to detect clusters and merge terms. This method generates an alternative representation of a value (“a key”) that encompasses only the most significant component of the string and groups different strings into “buckets” (or “bins”) based on identical keys. Researchers recognize the Fingerprint method as the most reliable method because it rarely produces false positives. Visual inspection of clustering result across the most frequent keywords confirmed high internal consistency, after which the algorithm was applied globally to minimize manual intervention. Consequently, we clustered “LC/MS” and “LCMS” as “LC-MS” and “UPLC-Q-TOF-MS” and “UPLC-QTOF-MS” as “UPLC-Q-TOF/MS”.

Although Key Collision methods process data quickly, they often prove too strict to accommodate variations among strings. Therefore, we conducted a secondary clustering using the Nearest Neighbor method with the Levenshtein distance function set to 1.0 and block characters set to 6 (Ham, 2013; CS&S, 2024). We then examined clusters manually because some groups, such as “vitamins”, incorporated terms like “vitamin D”, “vitamin A”, and “vitamin C”. Manual corrections were applied only to a limited number of explicitly identified cases where semantic overgeneralization was evident. Notable successful cases include “untargeted metabolomics” and “lipidomics,” which we manually unified with “untargeted-metabolomics” and “lipidomic”, respectively.

Finally, we merged terms with their most frequent forms, such as unifying “NMR” and “NMR spectroscopy” under Nuclear Magnetic Resonance, and “Gas Chromatography-Mass Spectrometry” under GC-MS. The proportion of manually modified terms was below 1% of the total keyword set.

The results of the automated clustering and the final harmonized keyword dataset are provided in Supplementary Files 6, 7, and 8.

# Data Analysis

We used OpenRefine to generate a bibliometrics file and obtain performance analysis and scientific mapping with Biblioshiny. We developed an R script to calculate the performance metrics of the most prolific authors directly reported by Scopus, from a record set obtained on March 21, 2025. We employed the same query, but the number of citations slightly increased. We conducted this analysis to avoid duplicating authors’ profiles resulting from different combinations of last names and first name initials.

Bibliometrix calculates scientific production by the number of authors in a country. For instance, an article with three Mexican authors represents three documents of Mexico, whereas we preferred an analysis that counts it as one. To address this, we developed an R script to calculate the metrics and employed Scimago Graphica for graphical representation (Hassan-Montero et al., 2022).

We then conducted a document analysis to address research questions concerning the most studied diseases and the predominance of specific databases. We created a dictionary to group related diseases into broader clusters. For example, we grouped “Alzheimer’s disease,” “Parkinson’s disease,” and “dementia” under the category of “Neurological_Neurodegenerative.” While we classified specific cancer types (e.g., breast cancer, glioblastoma) as “Cancer_Oncological.” We uploaded the constructed dictionary to Biblioshiny and filtered out terms unrelated to the primary focus.

We determined the frequency of disease mentions based on author keywords reported by Biblioshiny. We analyzed temporal trends to identify shifts in research focus, such as the prominence of studies related to the pandemic of severe acute respiratory syndrome (SARS-CoV-2) starting in 2020. We visualized the resulting data with a custom R script that generated temporal and frequency-based plots. We only considered disease keywords with more than five occurrences to ensure unified categories and avoid duplicated counts.

Additionally, we analyzed the Scopus metrics to rank journals based on their performance across all study areas. CiteScore 2023 (CS), based on a 4-year publication period, tallies the citations received in 2020-2023 of articles, reviews, conference papers, book chapters, and data articles published in 2020-2023 and divides them by the number of publications published under the same period. Researchers regard CiteScore as more open, free-access, and transparent than the Journal Impact Factor (Teixeira da Silva and Memon, 2017).

We considered the SCImago Journal Rank (SJR) of 2023, which evaluates a journal’s prestige based on the number of citations in related journals worldwide, allowing comparisons such as the Journal Impact Factor (Guz and Rushchitsky, 2009). Finally, we used the Source Normalized Impact per Paper (SNIP) of 2023, which quantifies contextual citation impact by weighting citations according to the total number of citations within a specific subject area. This metric emphasizes the number of references authors cite, the speed of the citation impact growth, and the scope of literature covered within a field (Moed, 2010).

# Multi-database validation

To verify the robustness and comprehensiveness of the results obtained from the Scopus dataset, we conducted an additional search in the Web of Science Core Collection (WoSCC) and PubMed on October 16, 2025. Although the retrieval dates differed, this did not represent a critical issue because equivalent search strategies – using the same set of keywords, Boolean operators, and a time frame limited to 2004-2024 – were adapted to the syntax requirements of each database. The inclusion and exclusion criteria applied in Scopus were consistently implemented in WoSCC and PubMed to ensure comparability

# References

Aria, M., and Cuccurullo, C. (2017). bibliometrix : An R-tool for comprehensive science mapping analysis. *J Informetr* 11, 959–975. doi: 10.1016/j.joi.2017.08.007

Brachet, P. (2023). Texmaker. Available at: https://www.xm1math.net/texmaker/ (Accessed February 11, 2024).

Derviş, H. (2020). Bibliometric Analysis using Bibliometrix an R Package. *Journal of Scientometric Research* 8, 156–160. doi: 10.5530/jscires.8.3.32

Guz, A. N., and Rushchitsky, J. J. (2009). Scopus: A system for the evaluation of scientific journals. *International Applied Mechanics* 45, 351–362. doi: 10.1007/s10778-009-0189-4

Hassan-Montero, Y., De-Moya-Anegón, F., and Guerrero-Bote, V. P. (2022). SCImago Graphica: a new tool for exploring and visually communicating data. *El Profesional de la información* 31. doi: 10.3145/epi.2022.sep.02

Linnenluecke, M. K., Marrone, M., and Singh, A. K. (2020). Conducting systematic literature reviews and bibliometric analyses. *Australian Journal of Management* 45, 175–194. doi: 10.1177/0312896219877678

Moed, H. F. (2010). Measuring contextual citation impact of scientific journals. *J Informetr* 4, 265–277. doi: 10.1016/j.joi.2010.01.002

Moral-Muñoz, J. A., Herrera-Viedma, E., Santisteban-Espejo, A., and Cobo, M. J. (2020). Software tools for conducting bibliometric analysis in science: An up-to-date review. *El Profesional de la Información* 29. doi: 10.3145/epi.2020.ene.03

Teixeira da Silva, J. A., and Memon, A. R. (2017). CiteScore: A cite for sore eyes, or a valuable, transparent metric? *Scientometrics* 111, 553–556. doi: 10.1007/s11192-017-2250-0
