# Supplementary figures and images for: A bibliometric and text-mining analysis of lipidomics and metabolomics in human disease
Source: Front Physiol. 2026 May 19;17:1727465. doi: 10.3389/fphys.2026.1727465 (PMC13225983; doi:10.3389/fphys.2026.1727465)

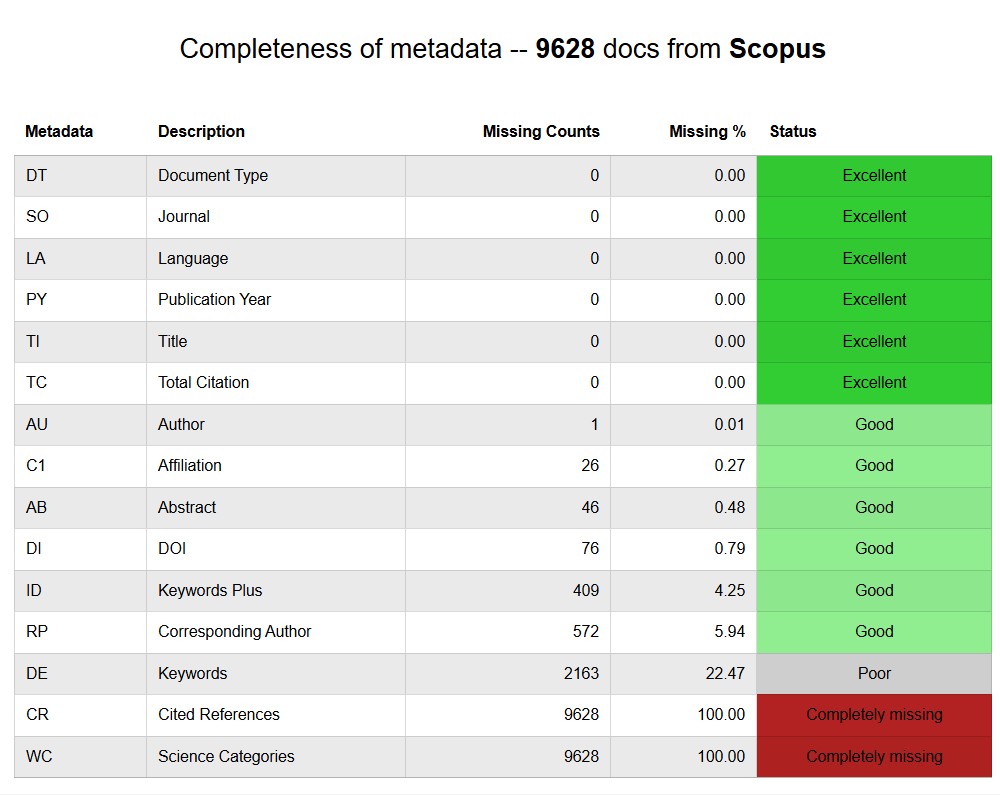

Supplement: Supplementary File 5 — Image 1.JPEG (Completeness.jpg) Biblioshiny report indicates the completeness of metadata. [file Image1.jpeg]
