# Supplementary material for: A bibliometric and text-mining analysis of lipidomics and metabolomics in human disease
Source: Front Physiol. 2026 May 19;17:1727465. doi: 10.3389/fphys.2026.1727465 (PMC13225983; doi:10.3389/fphys.2026.1727465)
Supplement: Supplementary File 17 — Table 9.DOCX (Supplementary_Material_WoSCC.docx) Validation analysis from WoSCC. [file Table9.docx]

Supplementary Material – WoSCC Validation

# Search Strategy

Search Query: (lipidome OR lipidomics OR metabolome OR metabolomics) AND (affliction OR ailment OR condition OR disease OR disorder OR illness OR infection OR malady OR pathology OR syndrome) AND (analysis OR assessment OR detection OR diagnosis OR determination OR evaluation OR examination OR identification OR investigation OR screening) AND (human* OR patient* OR clinical OR medicine OR biomedical OR healthcare OR hospital) NOT ("in vivo" OR nonhuman OR animal OR mice OR mouse OR rat* OR rodent OR canine OR pig OR swine OR "animal model") NOT (plant* OR vegetation OR crop* OR agriculture OR agronomy OR botany OR phytology OR phytochemical OR "plant-derived" OR "botanical extract" OR "phytotherapy" OR "plant model") (Topic) and 2004-2024 (Year Published) and English (Language) and Article (Document Type)

Following the filtration process, 7,418 results were obtained; however, 7333 were retained due to the fact that some articles were associated with 2025, whereas the analysis was conducted from 2004 to 2024.

# Supplementary Figures and Tables

Table 1. Summary of descriptive information on the dataset collection found from 2004 to 2024.

| Feature | Explanation | Count |
| --- | --- | --- |
| **Main Information about data** | | |
| Documents | Total number of scientific publications | 7333 |
| Sources | The frequency distribution of sources such as journals and books | 1687 |
| Annual growth rate % | The average increase in the number of documents over a year | 29.93 |
| Document average age | Average age of a document given in years | 5.3 |
| Average citations per doc | The average number of quotes in each article | 29.58 |
| **Document contents** | | |
| Keywords plus (ID) | Total number of words or phrases that frequently appear in the title of an article’s references | 11468 |
| Author’s keywords (DE) | Total number of keywords | 12886 |
| **Authors** | | |
| Authors | Total number of authors | 41714 |
| Authors of single-authored docs | The number of single authors per article | 76 |
| **Authors collaboration** | | |
| Single-authored docs | Total number of single-authored documents | 83 |
| Co-authors per doc | The average number of co-authors in each document | 10 |
| International Co-authorships % | The average number of articles with international collaboration | 28.61 |

Table 2. List of journals with the highest number of publications on the subject.

| Source | TP |
| --- | --- |
| Scientific Reports | 274 |
| Metabolites | 264 |
| Metabolomics | 235 |
| Journal of Proteome Research | 180 |
| PLoS ONE | 157 |
| International Journal of Molecular Sciences | 152 |
| Analytical Chemistry | 129 |
| Analytical and Bioanalytical Chemistry | 72 |
| Clinica Chimica Acta | 70 |
| Frontiers in Immunology | 70 |
| Journal of Pharmaceutical and Biomedical Analysis | 68 |
| Analytica Chimica Acta | 60 |
| Journal of Chromatography B | 60 |
| Frontiers in Molecular Biosciences | 55 |
| Journal of Chromatography A | 55 |
| Nutrients | 55 |
| Frontiers in Microbiology | 51 |
| Journal of Lipid Research | 49 |
| Cancers | 47 |
| Frontiers in Endocrinology | 43 |

TP = Total Publications

In this context, caution must be exercised, as Bibliometrix calculates scientific production based on the number of authors from a given country. To illustrate, an article authored by three Mexican scholars is counted as three documents from Mexico. However, our analysis prefers to count it as a single document.

Table 3. List of countries with the highest number of publications on the subject in our collection.

| Country | TP (Frequency of authors) |
| --- | --- |
| China | 12129 |
| United States | 9261 |
| Germany | 2519 |
| Italy | 2387 |
| Spain | 2325 |
| United Kingdom | 2275 |
| Netherlands | 1445 |
| Canada | 1421 |
| Japan | 1350 |
| France | 1344 |
| South Korea | 886 |
| Australia | 877 |
| Brazil | 797 |
| Sweden | 777 |
| India | 735 |
| Finland | 690 |
| Poland | 669 |
| Switzerland | 576 |
| Denmark | 454 |
| Singapore | 417 |

TP = Total Publications

Table 4. List of countries with the highest number of publications as corresponding on the subject in our collection.

| Country | CA | % | MCP | MCP/CA ratio |
| --- | --- | --- | --- | --- |
| China | 2143 | 29.2 | 236 | 0.110 |
| United States | 1297 | 17.7 | 368 | 0.284 |
| Italy | 354 | 4.8 | 108 | 0.305 |
| United Kingdom | 303 | 4.1 | 155 | 0.512 |
| Spain | 297 | 4.1 | 96 | 0.323 |
| Germany | 280 | 3.8 | 137 | 0.489 |
| Japan | 211 | 2.9 | 34 | 0.161 |
| Canada | 208 | 2.8 | 76 | 0.365 |
| Netherlands | 202 | 2.8 | 107 | 0.530 |
| France | 170 | 2.3 | 65 | 0.382 |
| India | 144 | 2 | 24 | 0.167 |
| Korea | 132 | 1.8 | 18 | 0.136 |
| Poland | 129 | 1.8 | 37 | 0.287 |
| Brazil | 124 | 1.7 | 29 | 0.234 |
| Sweden | 116 | 1.6 | 72 | 0.621 |
| Australia | 106 | 1.4 | 44 | 0.415 |
| Switzerland | 94 | 1.3 | 50 | 0.532 |
| Russia | 76 | 1 | 17 | 0.224 |
| Finland | 65 | 0.9 | 42 | 0.646 |
| Greece | 55 | 0.8 | 20 | 0.364 |

CA = Corresponding Author; MCP = Multiple Country Publication

Table 5. List of countries with the highest collaboration.

| Countries | Frequency |
| --- | --- |
| China – United States | 194 |
| United States – United Kingdom | 164 |
| United States – Germany | 133 |
| United States – Canada | 99 |
| Germany – United Kingdom | 94 |
| United Kingdom – Netherlands | 89 |
| Germany – Netherlands | 85 |
| United States – Italy | 81 |
| United States – Netherlands | 80 |

Table 6. Publications with the highest number of citations

| Title | Source | Article Type | TC | TCY | NTC |
| --- | --- | --- | --- | --- | --- |
| HMDB 4.0: the human metabolome database for 2018 | Nucleic Acids Research | Method | 3578 | 447.25 | 83.08 |
| HMDB: the Human Metabolome Database | Nucleic Acids Research | Method | 2502 | 131.68 | 12.26 |
| Procedures for large-scale metabolic profiling of serum and plasma using gas chromatography and liquid chromatography coupled to mass spectrometry | Nature Protocols | Method | 2439 | 162.60 | 20.52 |
| Gene Set Knowledge Discovery with Enrichr | Current Protocols | Method | 1917 | 383.4 | 83.29 |
| Gut microbiome structure and metabolic activity in inflammatory bowel disease | Nature Microbiology | Original Research | 1341 | 191.57 | 38.16 |


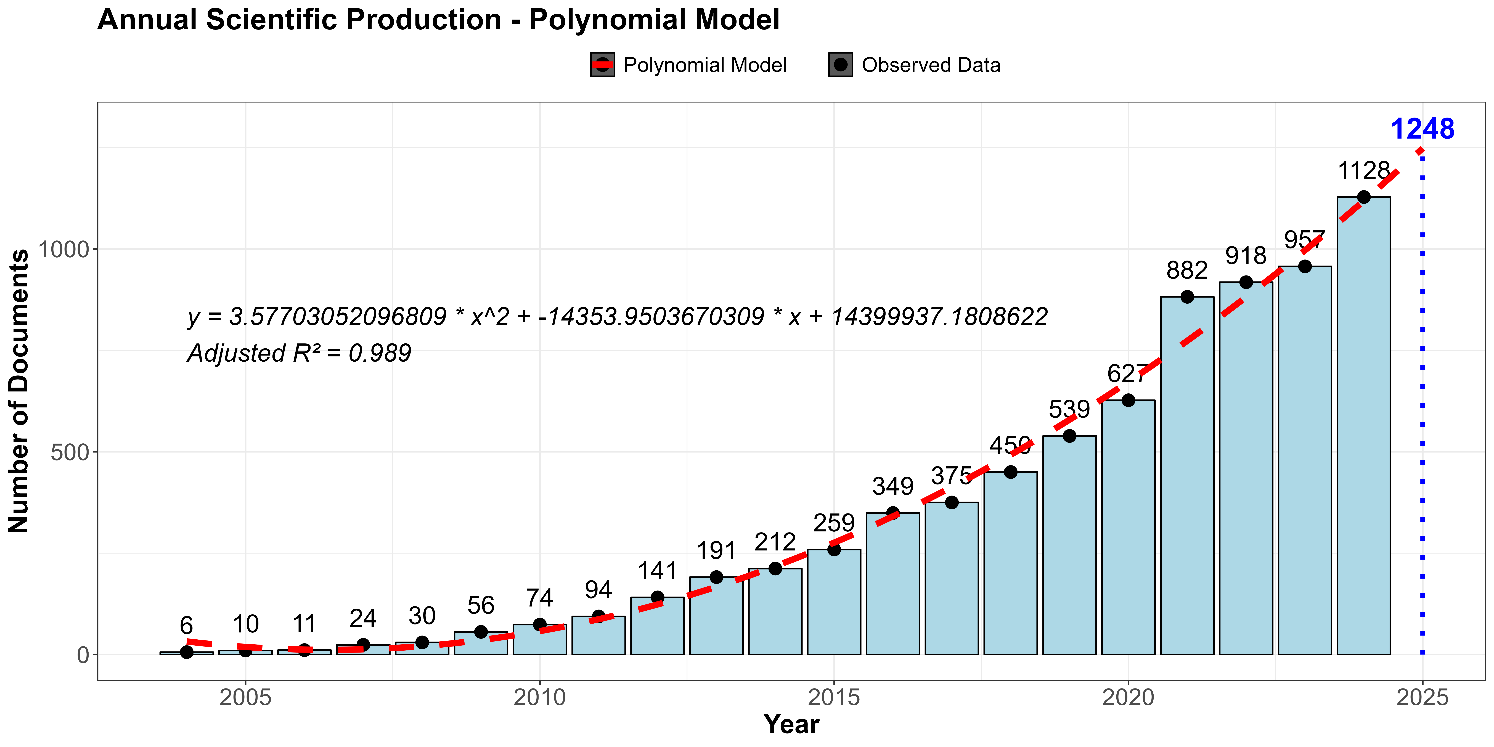


Figure 1. Annual distribution of scientific publications and polynomial forecast for 2025 in lipidomics and metabolomics for disease diagnosis.


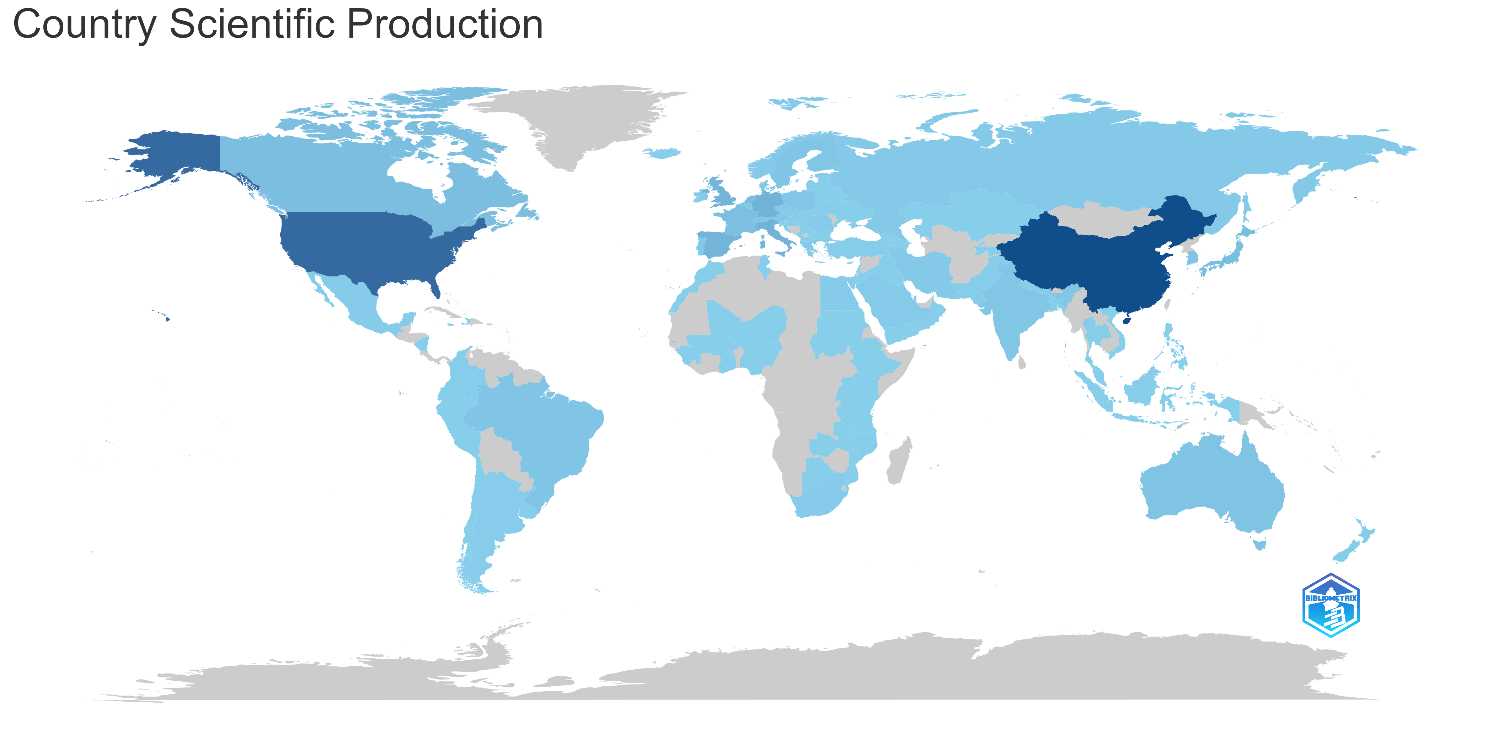


Figure 2. Scientific production by country. Countries in darker colors are more productive.


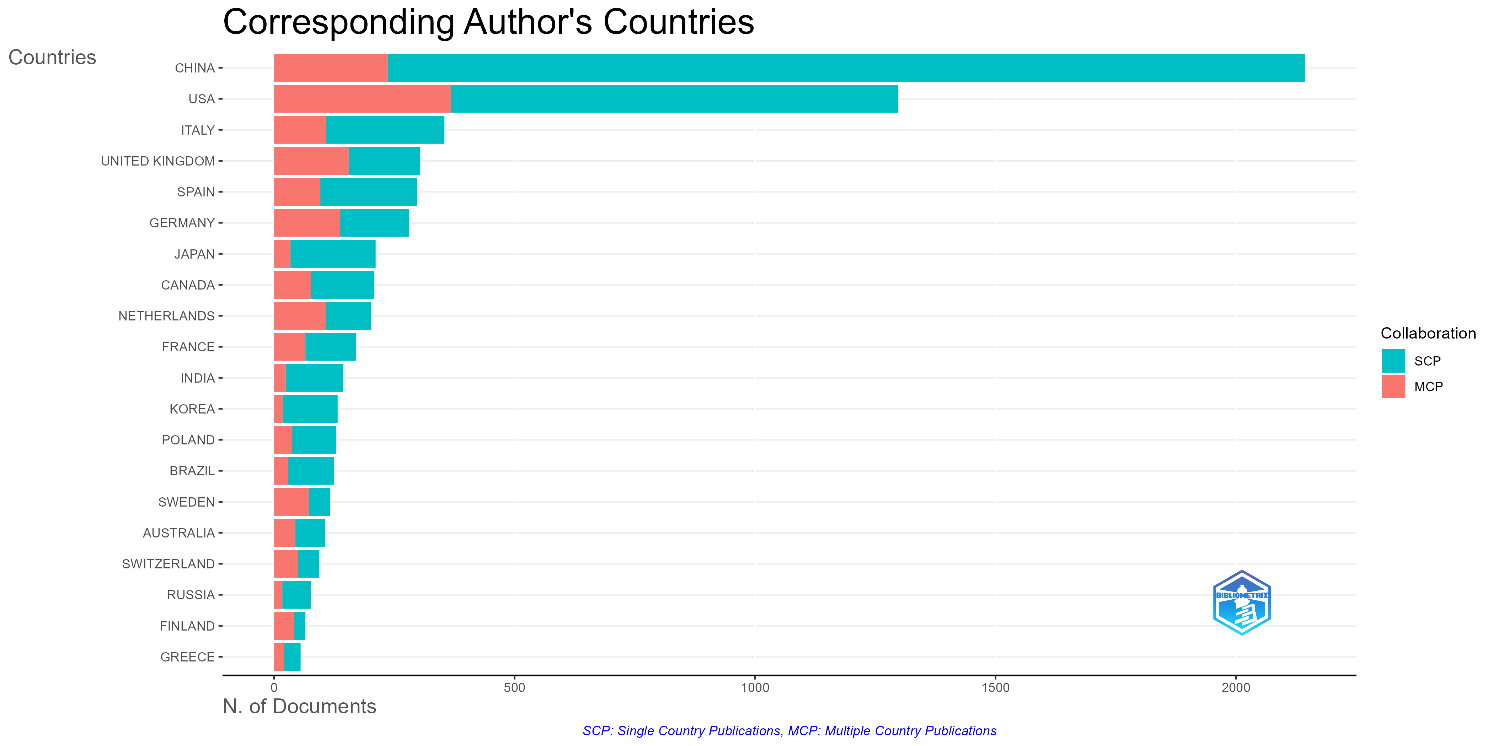


Figure 3. International collaboration by country. Classification goes as multiple country publications (MCPs) in red and at higher frequency, single country publications (SCPs) in blue.


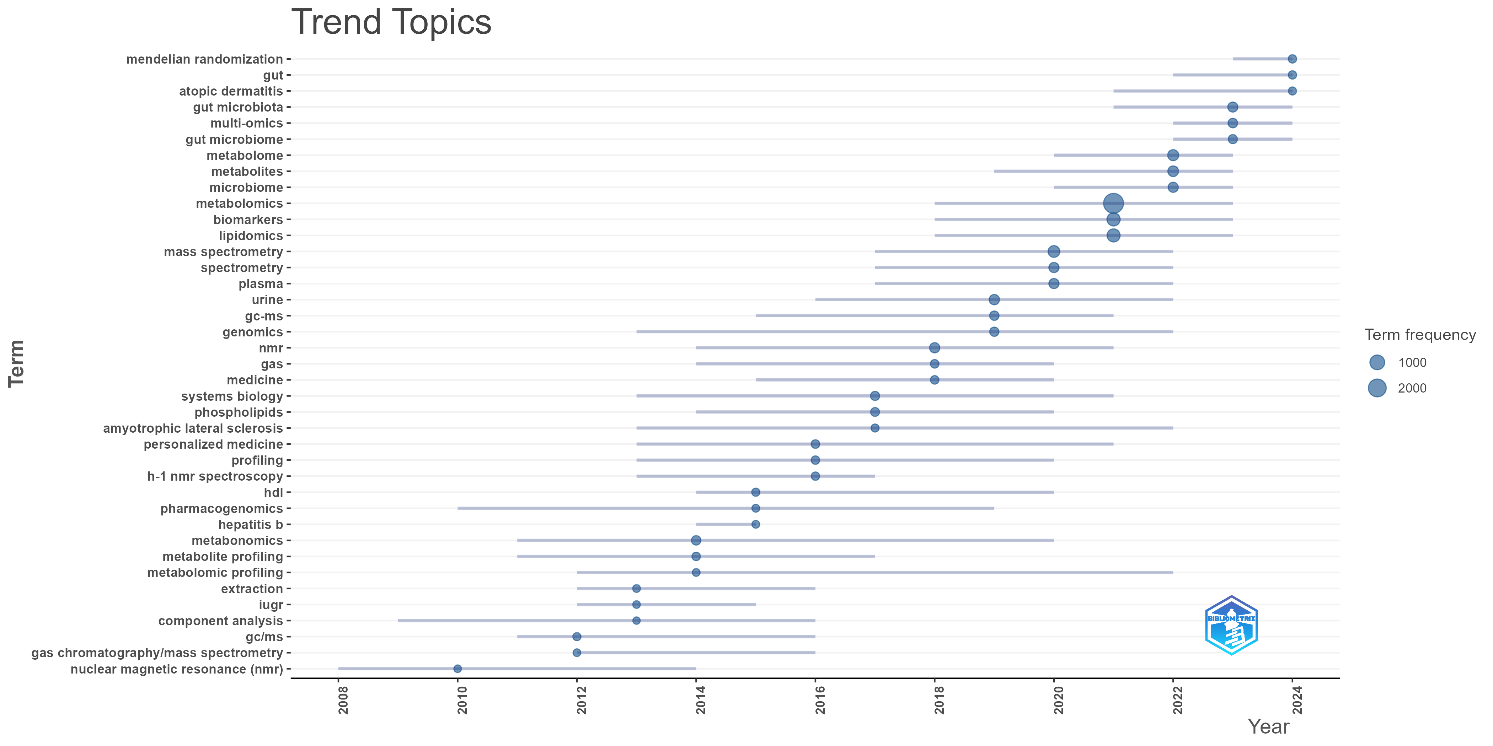


Figure 4. Trending topics. On the x-axis, we have the time span in years, and on the y-axis, the list of trending topics. The size of the circle indicates the frequency of the term, the position of the circle marks the year with the most publications on that topic, and the lines define how long that term has been present.
