# Supplementary material for: A bibliometric and text-mining analysis of lipidomics and metabolomics in human disease
Source: Front Physiol. 2026 May 19;17:1727465. doi: 10.3389/fphys.2026.1727465 (PMC13225983; doi:10.3389/fphys.2026.1727465)
Supplement: Supplementary File 18 — Table 10.DOCX (Supplemntary_Material_PubMed.docx) Validation analysis from PubMed. [file Table10.docx]

Supplementary Material – PubMed Validation

# Search Strategy

Search Query: ( ( (lipidome[Title/Abstract] OR lipidomics[Title/Abstract] OR metabolome[Title/Abstract] OR metabolomics[Title/Abstract]) AND (affliction[Title/Abstract] OR ailment[Title/Abstract] OR condition[Title/Abstract] OR disease[Title/Abstract] OR disorder[Title/Abstract] OR illness[Title/Abstract] OR infection[Title/Abstract] OR malady[Title/Abstract] OR morbus[Title/Abstract] OR pathology[Title/Abstract] OR syndrome[Title/Abstract]) AND (analysis[Title/Abstract] OR assessment[Title/Abstract] OR detection[Title/Abstract] OR diagnosis[Title/Abstract] OR determination[Title/Abstract] OR evaluation[Title/Abstract] OR examination[Title/Abstract] OR identification[Title/Abstract] OR investigation[Title/Abstract] OR screening[Title/Abstract]) AND (human*[Title/Abstract] OR patient*[Title/Abstract] OR clinical[Title/Abstract] OR medicine[Title/Abstract] OR biomedical[Title/Abstract] OR healthcare[Title/Abstract] OR hospital[Title/Abstract]) ) NOT ("in vivo"[Title/Abstract] OR nonhuman[Title/Abstract] OR animal[Title/Abstract] OR mice[Title/Abstract] OR mouse[Title/Abstract] OR rat*[Title/Abstract] OR rodent[Title/Abstract] OR canine[Title/Abstract] OR pig[Title/Abstract] OR swine[Title/Abstract] OR "animal model"[Title/Abstract]) NOT (plant*[Title/Abstract] OR vegetation[Title/Abstract] OR crop*[Title/Abstract] OR agriculture[Title/Abstract] OR agronomy[Title/Abstract] OR botany[Title/Abstract] OR phytology[Title/Abstract] OR phytochemical[Title/Abstract] OR "plant-derived"[Title/Abstract] OR "botanical extract"[Title/Abstract] OR "phytotherapy"[Title/Abstract] OR "plant model"[Title/Abstract]) ) AND ("2004/01/01"[Date - Publication] : "2024/12/31"[Date - Publication]) AND English[lang] AND humans[MeSH Terms].

Following the filtration process, 5,185 results were obtained; however, 5096 were retained due to the fact that some articles were associated with 2025, whereas the analysis was conducted from 2004 to 2024.

# Supplementary Figures and Tables

Table 1. Summary of descriptive information on the dataset collection found from 2004 to 2024.

| Feature | Explanation | Count |
| --- | --- | --- |
| **Main Information about data** | | |
| Documents | Total number of scientific publications | 5096 |
| Sources | The frequency distribution of sources such as journals and books | 1261 |
| Annual growth rate % | The average increase in the number of documents over a year | 27.58 |
| Document average age | Average age of a document given in years | 5.37 |
| Average citations per doc | The average number of quotes in each article | NA |
| **Document contents** | | |
| Keywords plus (ID) | Total number of words or phrases that frequently appear in the title of an article’s references | 12527 |
| Author’s keywords (DE) | Total number of keywords | 12527 |
| **Authors** | | |
| Authors | Total number of authors | 26817 |
| Authors of single-authored docs | The number of single authors per article | 101 |
| **Authors collaboration** | | |
| Single-authored docs | Total number of single-authored documents | 109 |
| Co-authors per doc | The average number of co-authors in each document | 8.74 |
| International Co-authorships % | The average number of articles with international collaboration | 22.17 |

Table 2. List of journals with the highest number of publications on the subject.

| Source | TP |
| --- | --- |
| Scientific Reports | 211 |
| International Journal of Molecular Sciences | 185 |
| Journal of Proteome Research | 127 |
| PLoS ONE | 126 |
| Metabolomics | 120 |
| Frontiers in Immunology | 76 |
| Clinica Chimica Acta | 68 |
| Journal of Pharmaceutical and Biomedical Analysis | 66 |
| Analytical Chemistry | 55 |
| Nutrients | 49 |
| Frontiers in Endocrinology | 48 |
| Journal of Chromatography B | 41 |
| Analytical and Bioanalytical Chemistry | 40 |
| Biomolecules | 40 |
| Journal of Translational Medicine | 35 |
| Frontiers in Cellular and Infection Microbiology | 34 |
| Journal of Lipid Research | 34 |
| Methods in Molecular Biology (Clifton, N.J.) | 33 |
| Analytica Chimica Acta | 30 |
| BMJ OPEN | 30 |

TP = Total Publications

In this context, caution must be exercised, as Bibliometrix calculates scientific production based on the number of authors from a given country. To illustrate, an article authored by three Mexican scholars is counted as three documents from Mexico. However, our analysis prefers to count it as a single document.

Table 3. List of countries with the highest number of publications on the subject in our collection.

| Country | TP (Frequency of authors) |
| --- | --- |
| China | 12741 |
| United States | 6898 |
| Italy | 2943 |
| Spain | 2652 |
| Germany | 2389 |
| Japan | 1380 |
| Netherlands | 1340 |
| France | 1315 |
| Canada | 1298 |
| Australia | 1162 |
| India | 914 |
| Brazil | 790 |
| South Korea | 704 |
| Poland | 700 |
| Sweden | 684 |
| United Kingdom | 582 |
| Finland | 535 |
| Switzerland | 519 |
| Denmark | 480 |
| Austria | 369 |

TP = Total Publications

Table 4. List of countries with the highest number of publications as corresponding on the subject in our collection.

| Country | CA | % | MCP | MCP/CA ratio |
| --- | --- | --- | --- | --- |
| China | 1173 | 23 | 114 | 0.097 |
| United States | 707 | 13.9 | 161 | 0.228 |
| Italy | 246 | 4.8 | 54 | 0.220 |
| Spain | 190 | 3.7 | 40 | 0.211 |
| Germany | 150 | 2.9 | 58 | 0.387 |
| Canada | 138 | 2.7 | 43 | 0.312 |
| Japan | 128 | 2.5 | 14 | 0.109 |
| Netherlands | 128 | 2.5 | 65 | 0.508 |
| India | 125 | 2.5 | 20 | 0.160 |
| France | 97 | 1.9 | 39 | 0.402 |
| Australia | 95 | 1.9 | 32 | 0.337 |
| South Korea | 79 | 1.6 | 9 | 0.114 |
| Sweden | 68 | 1.3 | 29 | 0.426 |
| Brazil | 66 | 1.3 | 17 | 0.258 |
| Poland | 65 | 1.3 | 21 | 0.323 |
| United Kingdom | 54 | 1.1 | 17 | 0.315 |
| Switzerland | 46 | 0.9 | 16 | 0.348 |
| Finland | 45 | 0.9 | 22 | 0.489 |
| Denmark | 39 | 0.8 | 22 | 0.564 |
| Greece | 38 | 0.7 | 12 | 0.316 |

CA = Corresponding Author; MCP = Multiple Country Publication

Table 5. List of countries with the highest collaboration.

| Countries | Frequency |
| --- | --- |
| China – United States | 104 |
| United States - Germany | 72 |
| United States - Canada | 62 |
| United States - Italy | 59 |
| Germany - Netherlands | 52 |
| United States - Netherlands | 42 |
| Italy - Germany | 39 |
| United States - Spain | 37 |
| Germany - Austria | 35 |


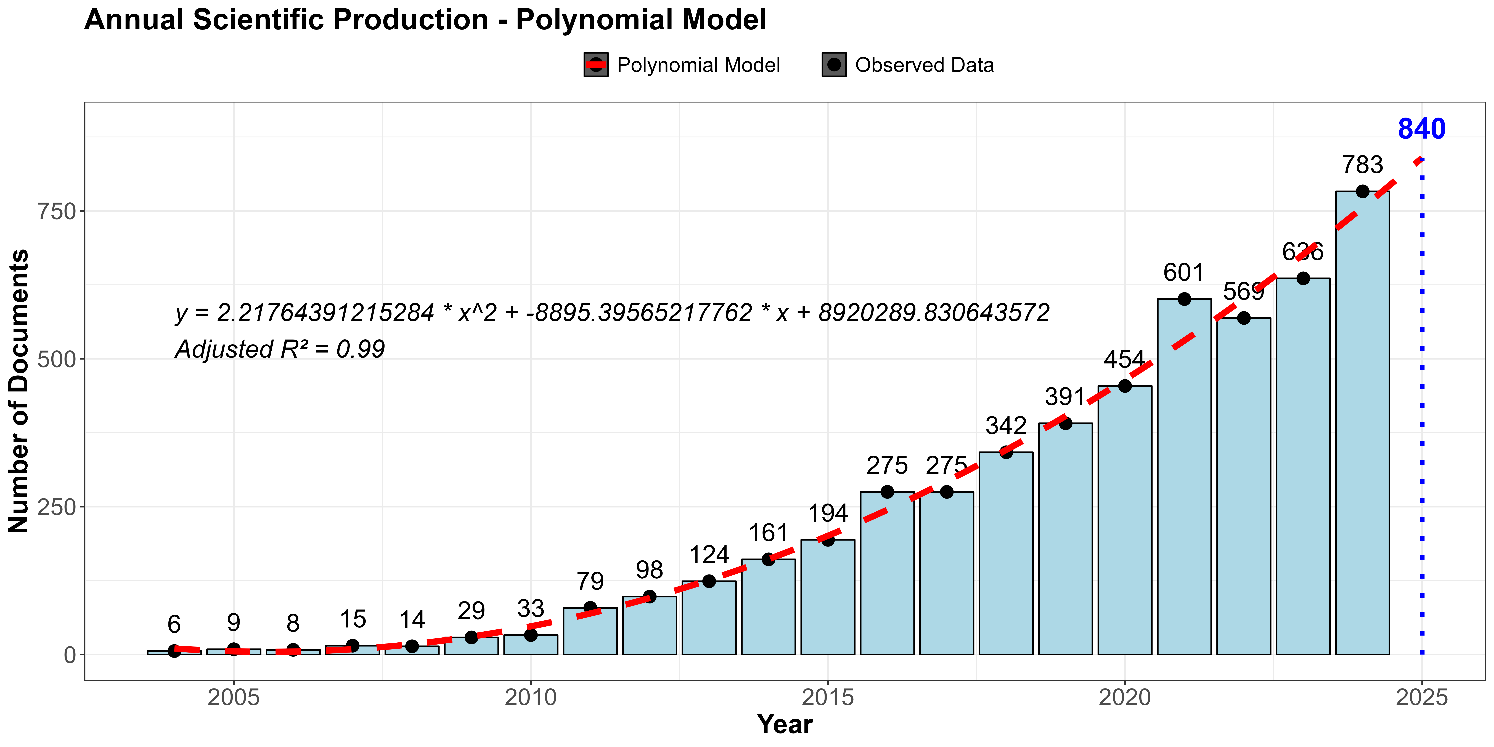


Figure 1. Annual distribution of scientific publications and polynomial forecast for 2025 in lipidomics and metabolomics for disease diagnosis.


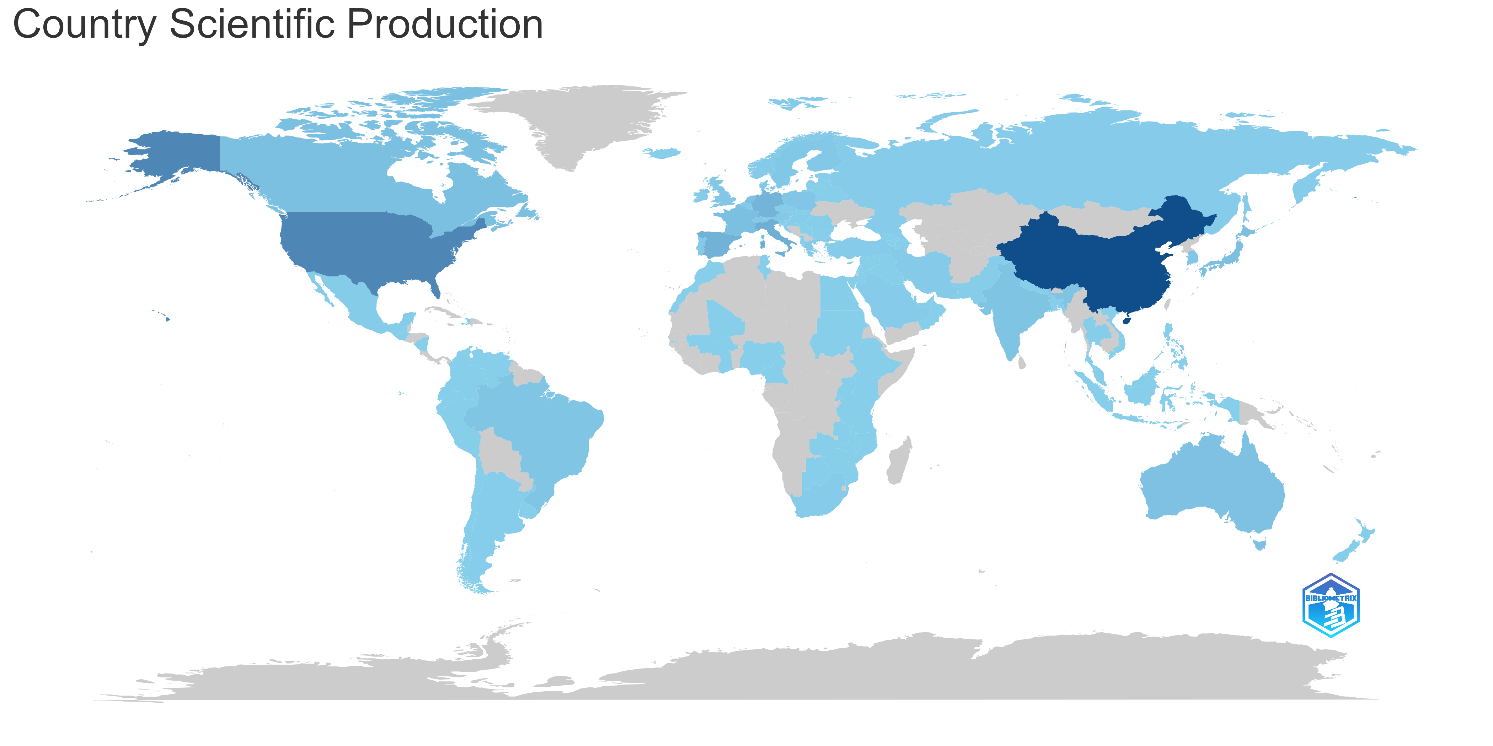


Figure 2. Scientific production by country. Countries in darker colors are more productive.


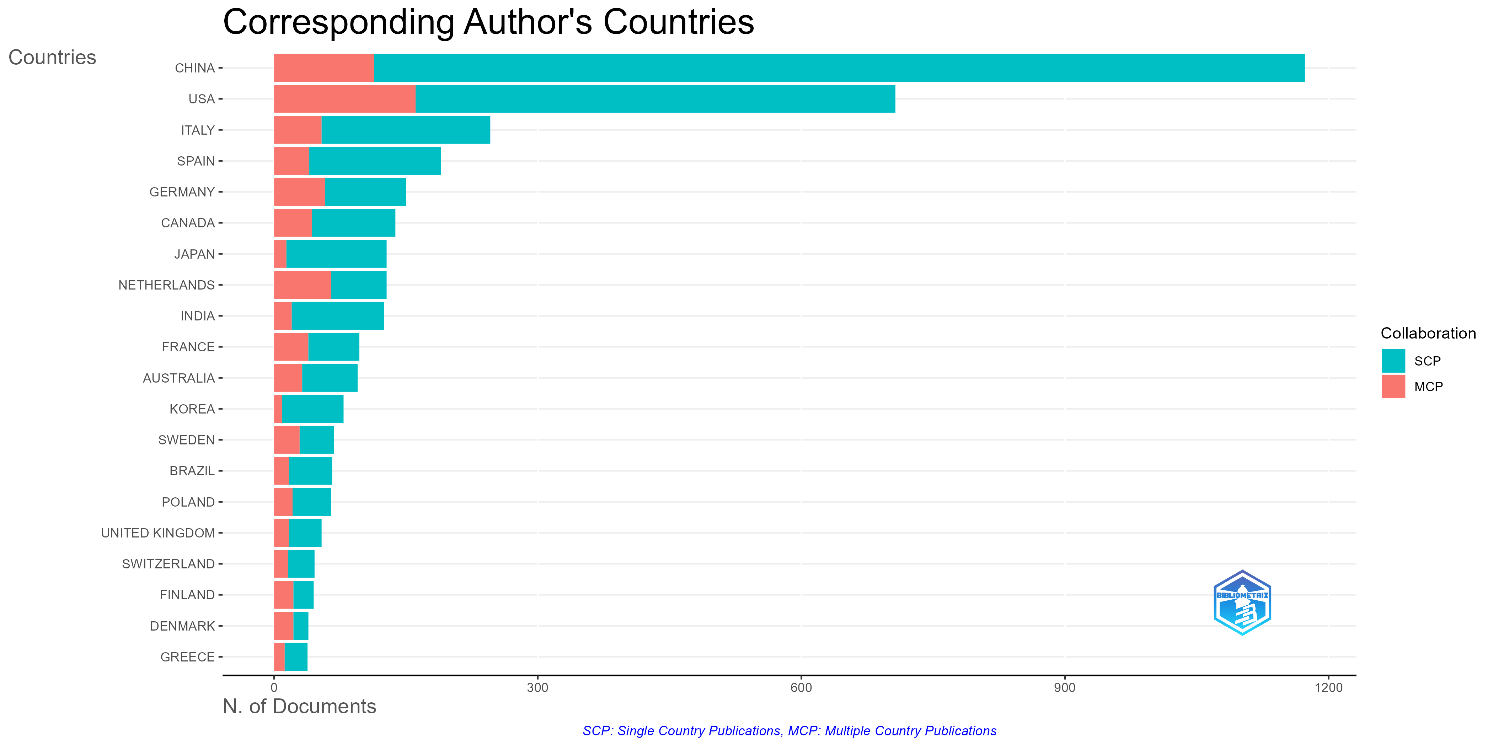


Figure 3. International collaboration by country. Classification goes as multiple country publications (MCPs) in red and at higher frequency, single country publications (SCPs) in blue.

The utilization of author keywords, such as Scopus, is not feasible due to the fact that, in the context of PubMed, keywords are MeSH terms. Consequently, the selection of keywords is based on the identification of unigrams present within the titles.


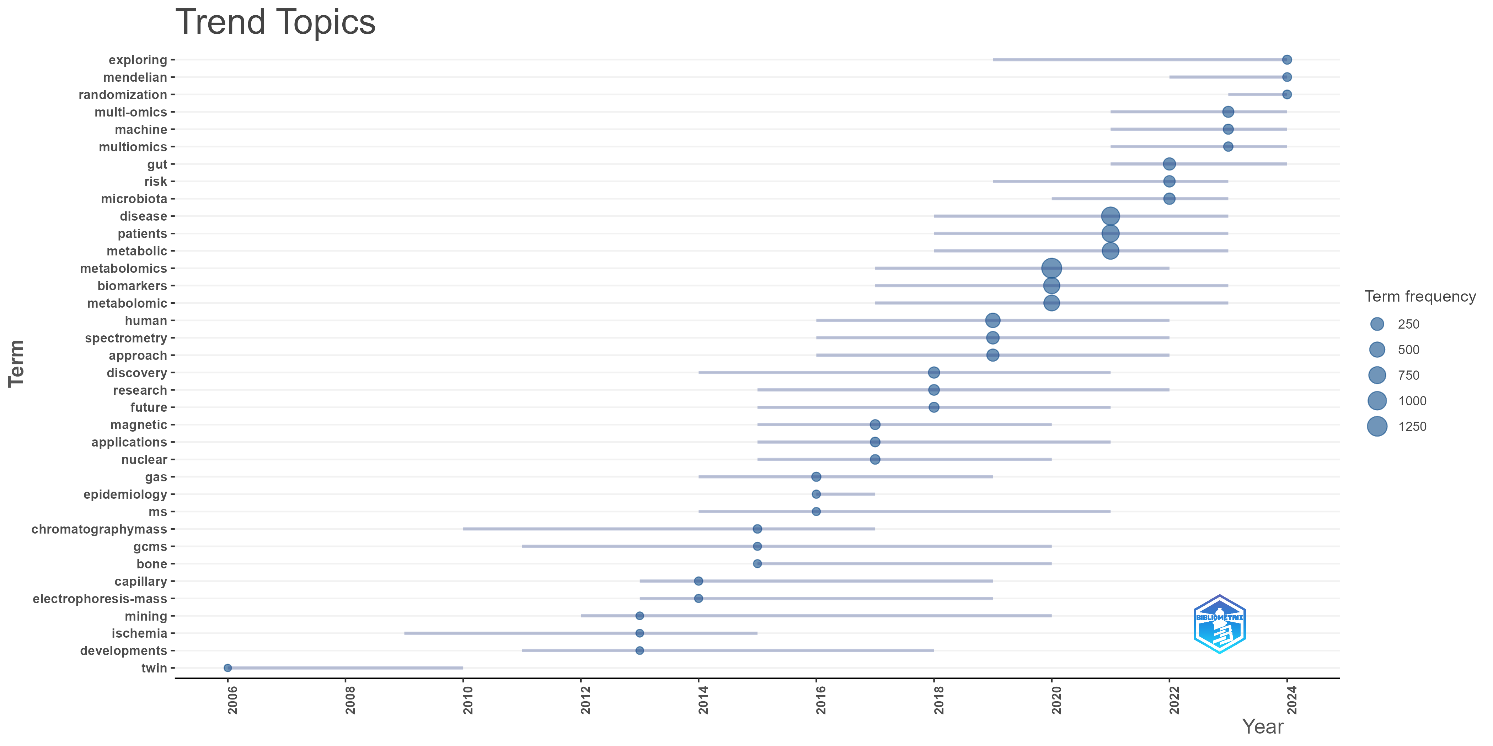


Figure 4. Trending topics. Here we used unigrams from the titles of the articles in the collection. On the x-axis, we have the time span in years, and on the y-axis, the list of trending topics. The size of the circle indicates the frequency of the term, the position of the circle marks the year with the most publications on that topic, and the lines define how long that term has been present.
